# Wnts Enhance Neurotrophin-Induced Neuronal Differentiation in Adult Bone-Marrow-Derived Mesenchymal Stem Cells via Canonical and Noncanonical Signaling Pathways

**DOI:** 10.1371/journal.pone.0104937

**Published:** 2014-08-29

**Authors:** Hung-Li Tsai, Wing-Ping Deng, Wen-Fu Thomas Lai, Wen-Ta Chiu, Charn-Bing Yang, Yu-Hui Tsai, Shiaw-Min Hwang, Perry F. Renshaw

**Affiliations:** 1 Graduate Institute of Medical Sciences, Taipei Medical University, Taipei, Taiwan; 2 Graduate Institute of Biomedical Materials and Engineering, Taipei Medical University, Taipei, Taiwan; 3 Graduate Institute of Clinical Medicine, Taipei Medical University, Taipei, Taiwan; 4 McLean Imaging Center, McLean Hospital/Harvard Medical School, Belmont, Massachusetts, United States of America; 5 Center for Nano-Tissue Engineering and Image Research, Taipei Medical University Hospital, Taipei, Taiwan; 6 Department of Neurosurgery, Taipei Medical University-Shuan-Ho Hospital, Taipei, Taiwan; 7 Department of Orthopedics, Taipei County Hospital, Taipei, Taiwan; 8 Bioresource Collection and Research Center, Food Industry Research and Development Institute, Hsinchu, Taiwan; 9 Department of Psychiatry and The Brain Institute, University of Utah, Salt Lake City, Utah, United States of America; University of Wisconsin-Madison, United States of America

## Abstract

Wnts were previously shown to regulate the neurogenesis of neural stem or progenitor cells. Here, we explored the underlying molecular mechanisms through which Wnt signaling regulates neurotrophins (NTs) in the NT-induced neuronal differentiation of human mesenchymal stem cells (hMSCs). NTs can increase the expression of *Wnt1* and *Wnt7a* in hMSCs. However, only Wnt7a enables the expression of synapsin-1, a synaptic marker in mature neurons, to be induced and triggers the formation of cholinergic and dopaminergic neurons. Human recombinant (hr)Wnt7a and general neuron makers were positively correlated in a dose- and time-dependent manner. In addition, the expression of synaptic markers and neurites was induced by Wnt7a and lithium, a glycogen synthase kinase-3β inhibitor, in the NT-induced hMSCs via the canonical/β-catenin pathway, but was inhibited by Wnt inhibitors and frizzled-5 (Frz5) blocking antibodies. In addition, hrWnt7a triggered the formation of cholinergic and dopaminergic neurons via the non-canonical/c-jun N-terminal kinase (JNK) pathway, and the formation of these neurons was inhibited by a JNK inhibitor and Frz9 blocking antibodies. In conclusion, hrWnt7a enhances the synthesis of synapse and facilitates neuronal differentiation in hMSCS through various Frz receptors. These mechanisms may be employed widely in the transdifferentiation of other adult stem cells.

## Introduction

Cells with neuronal characteristics appear to be generated *in vitro* from adult stem cells of putative mesodermal origin and can be isolated from various connective tissues, including bone marrow, umbilical cord blood, dermis, and adipose tissues [1], [2], [3], [4]. However, attempts to cause the transdifferentiation of adult bone marrow-derived cells into neural lineages *in vivo* have produced varied results. Some results showed the integration and differentiation of these cells in the brain [5], whereas others showed that the few cells capable of being engrafted into nervous tissues fused with endogenous cells.

Exogenic or allogenic progenitor cells are clinically required to serve as seeds of cellular repair for neural lesions. Among such candidates, adult bone marrow-derived mesenchymal stem cells (MSCs) deserve special attention because bone marrow harvesting is associated with fewer ethical debates than are embryonic cell sources. MSCs are multipotent stem cells that show osteogenic, chondrogenic, and adipogenic capacities in appropriate environments [6]. In addition, immunosuppression by MSCs has been observed *in vitro* and *in vivo* [7], [8]; thus, MSCs exhibit potential for clinical application. Previous studies demonstrated that either animal [2], [9], [10], [11] or human (h)MSCs [10], [11] can transdifferentiate into neuron-like cells that show neuronal markers such as NeuN, nestin, microtubule-associated protein-2 (MAP2), neuron-specific enolase (NSE), and neurofilament M (NFM). More recently, synaptophysin (SYP), a marker of neurites, was observed in rat MSCs *in vitro* [2]. The trigger for neuronal differentiation in previous studies was cytokines, including brain-derived neurotrophic factor (BDNF), nerve growth factor-β (NGF), neurotrophin (NT), fibroblast growth factor (FGF), retinoic acid (RA), and sonic hedgehog (Shh), or chemical reagents such as β-mercaptoethanol (BME), butylated hydroxyanisole (BHA), and dimethyl sulfoxide (DMSO). However, no neurite marker has been reported in hMSCs *in vitro*. The terminal neuronal differentiation of hMSCs for neuronal regeneration remains poorly understood.

Wnt plays an essential role in neuronal initiation and maturation in embryonic development. 19 secreted Wnt proteins have been found in humans and are categorized into 12 families. In the canonical pathway, Wnt ligands interact with Frizzled (Frz) receptors and co-receptors, low-density lipoprotein receptor-related protein (LRP)5/6, resulting in inhibition of glycogen synthase kinase (GSK)-3β. This leads to β-catenin accumulation in the cytoplasm and nuclear translocation. In nuclei, β-catenin associates with the T-cell factor (TCF) and lymphoid enhancer factor (LEF) to induce transcription of target genes [12]. Other than the canonical/β-catenin pathway, Wnts also activate the non-canonical/c-Jun N-terminal kinase (JNK) pathway [13], [14] or the non-canonical/calcium pathway to control cell behaviors [15], [16]. The canonical Wnt pathway was reported to trigger differentiation in neural progenitors in mice [17], [18]. Wnt3 stimulates axon branching and extends the growth cone size in proprioceptive NT3-responsive sensory neurons [19]. Wnt7a promotes the neurogenesis of cortical neural precursor cells [20] and synaptogenesis in cerebellar and hippocampal neurons in animals [21]. In β-catenin-independent Wnt signaling, Wnt7b activates signaling through disheveled (Dvl), Rac, and JNK in immature hippocampal neurons which show enhanced number and length of dendritic branching [22].

Attempts were made to understand Wnt regulation of osteogenesis or adipogenesis using hMSCs [23]. In addition, canonical Wnt was shown to augment the invasion and proliferation of hMSCs [24]. However, the effects of canonical or non-canonical Wnt signaling in neurogenic hMSCs are still little understood.

The goal of this study was to determine whether Wnt proteins can enhance neurotrophin's effect on neuronal differentiation of hMSCs, defining the pathways of signaling into neurite phenotypes and specific neuron types. Neurite formation and determination of specific neurons were examined by mRNA expression and immunocytochemistry. We conclusively demonstrated enhancement of neuronal differentiation in neurotrophin-induced hMSCs after cultivation with Wnts. Canonical and non-canonical Wnt signaling via varied receptors facilitated transdifferentiation into neuro-ectodermal lineages.

## Materials and Methods

### Ethics Statement

The protocols and informed consent form for bone marrow hMSC isolation were approved by the Taipei Medical University Joint Institutional Review Board (TMUH-03-08-12). The specimen donor was provided with an IRB-approved formal consent form describing sufficient information for that person to make an informed decision about his/her participation in this study. The formal consent form was signed by the subject before specimen collection.

### Isolation and cultivation of hMSCs

Bone marrow samples were collected from five consenting patients (age: 50–70) without endocrine disease in the Orthopedic Section of Taipei Medical University Hospital (Taipei, Taiwan). hMSCs were obtained using gradient centrifugation. Diluted samples were placed on Percoll gradients (1.073 g/mL) (GE Healthcare). Next, samples were fractionated using centrifugation, and the MSC-enriched interface layer was collected. Isolated hMSCs were mixed with hMSCs that were supplied by Cambrex, and both types of hMSCs were cultured in 10-cm dishes using Dulbecco's modified Eagle's medium with low glucose (DMEM/LG) (Invitrogen), 10% fetal bovine serum (FBS) (Invitrogen), and a 1% penicillin-streptomycin mixture (Invitrogen). These mixtures were cultured at 37°C in a humidified atmosphere with 5% CO_2_. The medium was refreshed three times per week, and cells were subcultured to confluence. All experiments were performed with cells from passages 3–6.

### Flow cytometric analysis

Confluent hMSCs (passages 3–6) were detached using a brief trypsin treatment. The cells were fixed with 4% formaldehyde and 100% ice-cold methanol. Then, 5×10^5^ cells were incubated with each mouse monoclonal primary antibody. These antibodies included mouse phycoerythrin (PE)-labeled anti-CD14 (1∶500; clone M5E2, cat#555398, BD Biosciences), fluorescein isothiocyanate (FITC)-labeled anti-CD34 (1∶500; clone 581/CD34, cat#555821, BD Bioscience), PE-labeled anti-CD44 (1∶500; clone 515, cat#550989, BD Bioscience), PE-labeled anti-CD73 (1∶500; clone AD2, cat#550257, BD Bioscience), FITC-labeled anti-CD105 (1∶500; clone SN6, cat#MCA1557F, AbD Serotec), PE-labeled anti-CD166 (1∶500; clone 3A6, cat#559263, BD Bioscience), and anti-stro1 (1∶500; clone STRO-1, cat#MAB1038, R&D Systems). After incubation of the primary antibodies, secondary FITC-labeled immunoglobulin G (IgG) antibodies (1∶100; Chemicon) were added to the group with unlabeled primary antibodies. Following a final wash, cells were resuspended in a 0.5 mL of buffer and analyzed on a Becton Dickinson FAC Scalibur (Becton Dickinson). Mouse PE-labeled IgG1, FITC-labeled IgG1, PE-labeled IgG2a, and IgM were used as negative controls.

### Neuronal transdifferentiation of hMSCs

To induce neuronal transdifferentiation, 10^5^ hMSCs (passages 3–6) in 6-well plates were treated with NTs consisting of 1% FBS, 10 ng/mL BDNF (Chemicon), 20 ng/mL NGF (Chemicon), and 5 µM RA (Sigma) at 37°C in a humidified atmosphere with 5% CO_2_, and the medium was refreshed three times per week. After 7 days of neurogenic differentiation, human recombinant (hr)Wnt1 (Peprotech), hrWnt3a (R&D Systems), hrWnt5a (R&D Systems), hrWnt7a (R&D Systems), and LiCl (Sigma) were added at the indicated concentrations (0.1∼2 µg/mL or 1∼4 mM) at various times (0∼48 h) for differentiation. In addition, hrWnt7a or LiCl in DMEM/LG with 10% FBS was incorporated into hMSCs as the control to confirm that Wnt signaling had no effect on neurogenesis. Wnt7a signaling was inhibited by recombinant human dickkopf-1 (DKK1) (R&D Systems), secreted frizzle-related protein-4 (sFRP4) (R&D Systems), anti-human polyclonal Frz5 (cat#AF1617, R&D Systems), anti-mouse Frz9 monoclonal antibodies (clone 291004, cat#MAB2440, R&D Systems), and SP600125 (Santa Cruz). Furthermore, 24 h before the addition of hrWnt7a, NT-induced hMSCs were treated with hrDKK1 (0.5 µg/mL), sFRP4 (2.5 µg/mL), anti-human Frz5 (1 µg/mL), anti-mouse Frz9 antibodies (1 µg/mL), or SP600125 (15 µM). Next, Wnt7a was incubated with the inhibitors in NT-induced hMSCs for 48 h.

### RNA isolation and quantitative reverse-transcription polymerase chain reaction (qPCR)

Total RNA from the 10^5^ hMSCs (passages 3–6) was extracted using the TRIzol reagent (Invitrogen). cDNA synthesis was performed using a SuperScript III system (Invitrogen). The qPCR was carried out on a LightCycler 480 system (Roche Diagnostics) using an LC-FastStart DNA Master SYBR Green I mix (Roche). In addition, 5 µL of each cDNA was rapidly mixed with 1 µL of forward and reverse primers and 13 µL of LC-FastStart DNA Master SYBR Green I mix. The amplification profile was as follows: enzyme activation at 95°C for 10 min; and annealing at 95°C for 10 s, 60°C for 5 s, and 72°C for 15 s. The specificity of the PCR products was determined by a melting curve analysis. The forward and reverse primers of the human genes were designed using LightCycler Probe Design Software 2 (Roche), and sequences are shown in table 1. Results are expressed relative to the housekeeping gene, glyceraldehyde 3-phosphate dehydrogenase (*GAPDH*).

**Table 1:** Primers for the qPCR.

Gene	Sequence (forward; reverse)	Product length
*GAPDH* (glyceraldehyde 3-phosphate dehydrogenase)	5′-CGACCACTTTGTCAAGCTCA-3′ 5′-AGGGGTCTACATGGCAACTG-3′	228
*MAP2* (microtubule-associated protein 2)	5′-TTGGTGCCGAGTGAGAAGAA-3′ 5′-GGTCTGGCAGTGGTTGGTTAA-3′	100
*Nestin*	5′-AAGAGAGCATAGAGGCAGTAA-3′ 5′-GAGATTTCAGTGTTTCCAGGT-3′	93
*Neurotublin* (neuron-specific class III beta-tubulin)	5′-CTCTTCTCACAAGTACGTGC-3′ 5′-CCTGAAGAGATGTCCAAAGG-3′	97
*SYTG* (synaptotagmins 1)	5′-TATTTGAGGAAGCAACTGAACAGG-3′ 5′-CACACACACACACACGGA-3′	87
*SYN* (synapsin 1)	5′-GCAAACTCCACCCATCTT-3′ 5′-ACACAGACACCACAGCA-3′	85
*BSN* (bassoon)	5′-CCAAGGGTTTGCCAGATTGTA-3′ 5′-GCTCTTGAAGAAATGAAGAGAGGTA-3′	115
*ChAT* (choline acetyltransferase)	5′-CCGGTTTGTCCTCTCCACTA-3′ 5′-ATACCCATTTGGGACCACAG-3′	82
*DBH* (dopamine beta-hydroxylase)	5′-ATATCTCCGCCTGGAAGTTCA-3′ 5′-TGTGTAGTACAAGCGGATGC-3′	85
*DVL* (dishevelled 1)	5′-CCACCCTGAACCTCAACAGT-3′ 5′-CCTTCACTCTGCTGACTCCC-3′	202
*LEF* (lymphoid enhancer-binding factor 1)	5′-AAGGAGCAGGAGCCAAA-3′ 5′-CTCAGCAACGACATTCGC-3′	93
*Wnt1*	5′-AACAGCGGCGTCTGATAC-3′ 5′-GCGGAGGTGATAGCGAAG-3′	199
*Wnt3a*	5′-ATGAACCGCCACAACAAC-3′ 5′-TTCTCCACCACCATCTCC-3′	188
*Wnt5a*	5′-GGGAGGTTGGCTTGAACATA-3′ 5′-GAATGGCACGCAATTACCTT-3′	141
*Wnt7a*	5′-TGGCTTCTCCTCAGTGGTAG-3′ 5′-CCTTCTCCTATGACGATGATGG-3′	123
*Wnt7b*	5′-TATCCCAGAGAGCAAAGTG-3′ 5′-TGTGTTAGTGCCGAGAATC-3′	204

### Immunocytochemistry

hMSCs (passages 3–6) were fixed with 4% paraformaldehyde for 10 min, and were permeabilized with 0.02% Triton X-100 for 10 min, followed by blocking with 5% FBS for 1 h and incubation with primary antibodies for at least 1 h. The primary antibodies were as follows: rabbit anti-MAP2 polyclonal antibodies (1∶500; cat#AB5622, Chemicon); rabbit anti-synapsin-1 (SYN1) polyclonal antibodies (1∶500; cat#AB1543, Chemicon); mouse anti-choline acetyltransferase (ChAT) monoclonal antibodies (1∶500; clone 1E6, cat#MAB305, Chemicon); and rabbit anti-dopamine β-hydroxylase (DBH) polyclonal antibodies (1∶500; cat#AB1585, Chemicon); and mouse anti-β-catenin monoclonal antibodies (1∶500; clone 5H10, cat#MAB2081, Chemicon,). Cells were then incubated for 1 h with secondary antibodies, goat anti-mouse IgG:Dylight 488 (1∶100; Serotec), or sheep anti-rabbit IgG:Dylight 649 (1∶100; Serotec). The nuclear stain was 4',6-diamidino-2-phenylindole (DAPI) (Chemicon). Images were obtained using fluorescent microscopy (Hamamatsu EM-CCD Camera C9100-13). For β-catenin staining, cells with projections exceeding 30 µm were considered neurite-positive cells. The cell area and neurite length were calculated using ImageJ software. At least 1000 cells from 10∼15 viewing fields per group were used to calculate percentages of cells. The human SH-SY5Y neuroblastoma cell line was used as the positive control, and the human MG63 osteosarcoma cell line was used as the negative control. Mouse IgG1 antibodies (Chemicon) were used as the isotype control. All control results are shown in Figure S1.

### Western blotting

After treatment, hMSCs (passages 3–6) were trypsinized and dissolved in the M-PER Mammalian Protein Extraction Reagent (Thermo Scientific), and then centrifuged for 15 min at 12,000 rpm and 4°C. The upper fluid, containing total protein, was extracted. The extracted protein was denatured for 5 min at 95°C and loaded on a 10% SDS–PAGE gel. After electrophoresis, denatured proteins were transferred onto polyvinylidene fluoride membranes (Amersham Biosciences). The membrane was blocked for 30 min in tris-buffered saline with 0.05% Tween-20 (TBS-T) containing 5% bovine serum albumin and incubated overnight with primary antibodies in TBS-T containing 2% nonfat milk. The peroxidase-conjugated affinpure anti-mouse or anti-rabbit IgG (Jackson ImmunoResearch) was added for 1 h. Bands were visualized on film (Hyperfilm ECL, Amersham Pharmacia) by using the ECL Plus kit (Amersham Pharmacia). The primary antibodies were rabbit anti-MAP2 polyclonal antibodies (1∶1000; cat#AB5622, Chemicon); rabbit anti-SYN1 polyclonal antibodies (1∶1000; cat#AB1543, Chemicon); mouse anti-ChAT monoclonal antibodies (1∶1000; clone 1E6, cat#MAB305, Chemicon); rabbit anti-DBH polyclonal antibodies (1∶1000; cat#AB1585, Chemicon); and rabbit anti-GAPDH polyclonal antibodies (1∶10000; cat#ABS16, Chemicon). Electrophoresis and transfer materials were purchased from Bio-Rad.

### Statistical analysis

Data are presented as mean values ± SD of all experiments or a representative result of three or more experiments. Quantitative data were analyzed using SigmaPlot 9.0 or SPSS software by conducting either the Student's *t* test or a one-way ANOVA. A value of *p*<0.05 (*, ^#^, ^1^, ^2^, ^3^, ^4^, ^5^ or ^6^) and *p*<0.01 (**, ^##^, ^1″^, ^2″^, ^3″^, ^4″^, ^5″^ or ^6″^) indicated significance.

## Results

### Characterization of bone-marrow-derived human mesenchymal stem cells

**Figure 1:**
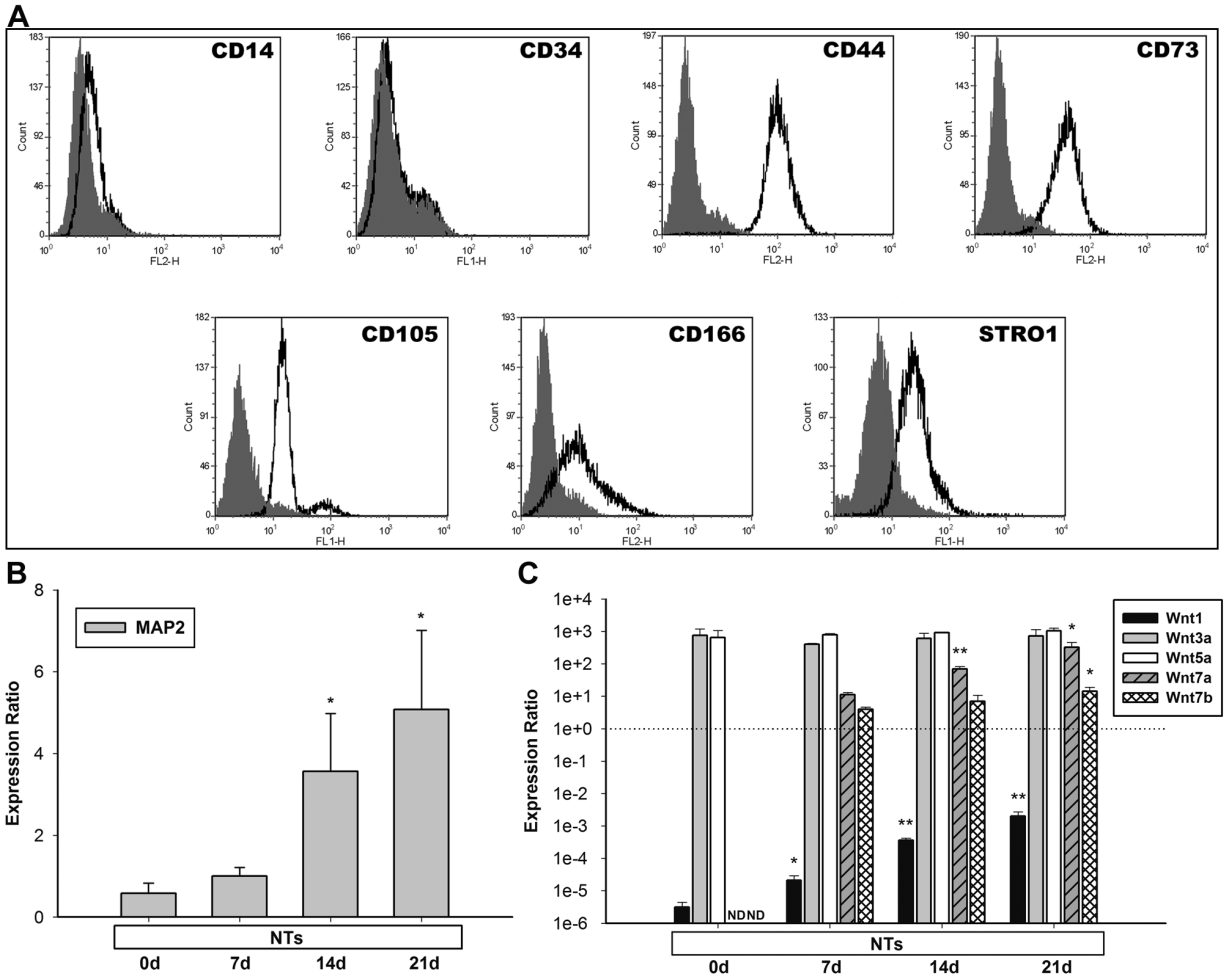
Flow cytometric analysis and Wnt profiles of hMSCs by the induction of NTs. (A) Bone marrow-derived hMSCs were analyzed following four cell passages. hMSCs were positive for CD44, CD73, CD105, CD166, and Stro-1, and negative for CD14 and CD34. The solid curves indicate each type of antibody, and the filled curves indicate mouse IgG as the negative control. (B) mRNA levels of *MAP2* were quantified on days 7, 14, and 21 during stimulation with NTs. NTs significantly increased *MAP2* levels on days 14 and 21. Untreated hMSCs served as the control. (C) mRNA levels of *Wnt1*, *Wnt3a*, *Wnt5a*, *Wnt7a*, and *Wnt7b* were quantified on days 7, 14, and 21 during stimulation with NTs. NTs increased the expression of *Wnt1* and induced expressions of *Wnt7a* and *Wnt7b*. * *p*<0.05, ** *p*<0.01 (i.e., treated vs. control in the Wnt1, Wnt3a, and Wnt5a groups; NTs at 14 and 21 days vs. NTs at 7 days in the Wnt7a and Wnt7b groups). Data are presented as the mean ± SD of one triplicate experiment that was representative of three independent experiments. * *p*<0.05, ** *p*<0.01 (i.e., treated vs. control). ND, not determined.

The human bone-marrow-derived MSCs were characterized using cell surface markers by performing flow cytometric analysis (Fig. 1A). The MSCs tested positively for hMSC-specific cell type markers, such as CD44, CD73, CD105, CD166, and Stro1, as indicated in previous reports [6], [25], and the hematopoietic-stem-cell (HSC)-specific cell type markers CD14 and CD34 were negative in isolated hMSCs after 3 passages [6], [25]. The data revealed that none of the isolated cells were HSCs or blood cells.

### Expression profile of Wnt family genes during the neuronal differentiation of human mesenchymal stem cells

We confirmed the neuronal induction of NTs by using a neuronal marker, *MAP2*, in the cultivation system (Fig. 1B), and then determined the roles of Wnts during hMSC neurogenesis. A qPCR was used to examine the mRNA expression of *Wnt1*, *Wnt3a*, *Wnt5a*, *Wnt7a*, and *Wnt7b* in hMSCs cultured with NTs. Canonical *Wnt3a* and non-canonical *Wnt5a* exhibited sustained expression with no significant changes during neuronal transdifferentiation (Fig. 1C). NTs seemed not to control these 2 Wnt genes in neurogenic hMSCs. Canonical *Wnt1* exhibited relatively low expression in both the untreated control and NT-induced groups compared with the other 4 Wnt mRNAs at each time point (Fig. 1C). The mRNA levels of *Wnt7a* and *Wnt7b* were not detectable in untreated hMSCs, but were expressed significantly following NT induction during 3 periods (Fig. 1C). The expression of both *Wnt7a* and *Wnt7b* significantly increased over time. These results indicated that Wnt7a and Wnt7b play roles in the neuronal differentiation of hMSCs.

### Neurogenic effects of various Wnt treatments on neurotrophin-induced human mesenchymal stem cells

**Figure 2:**
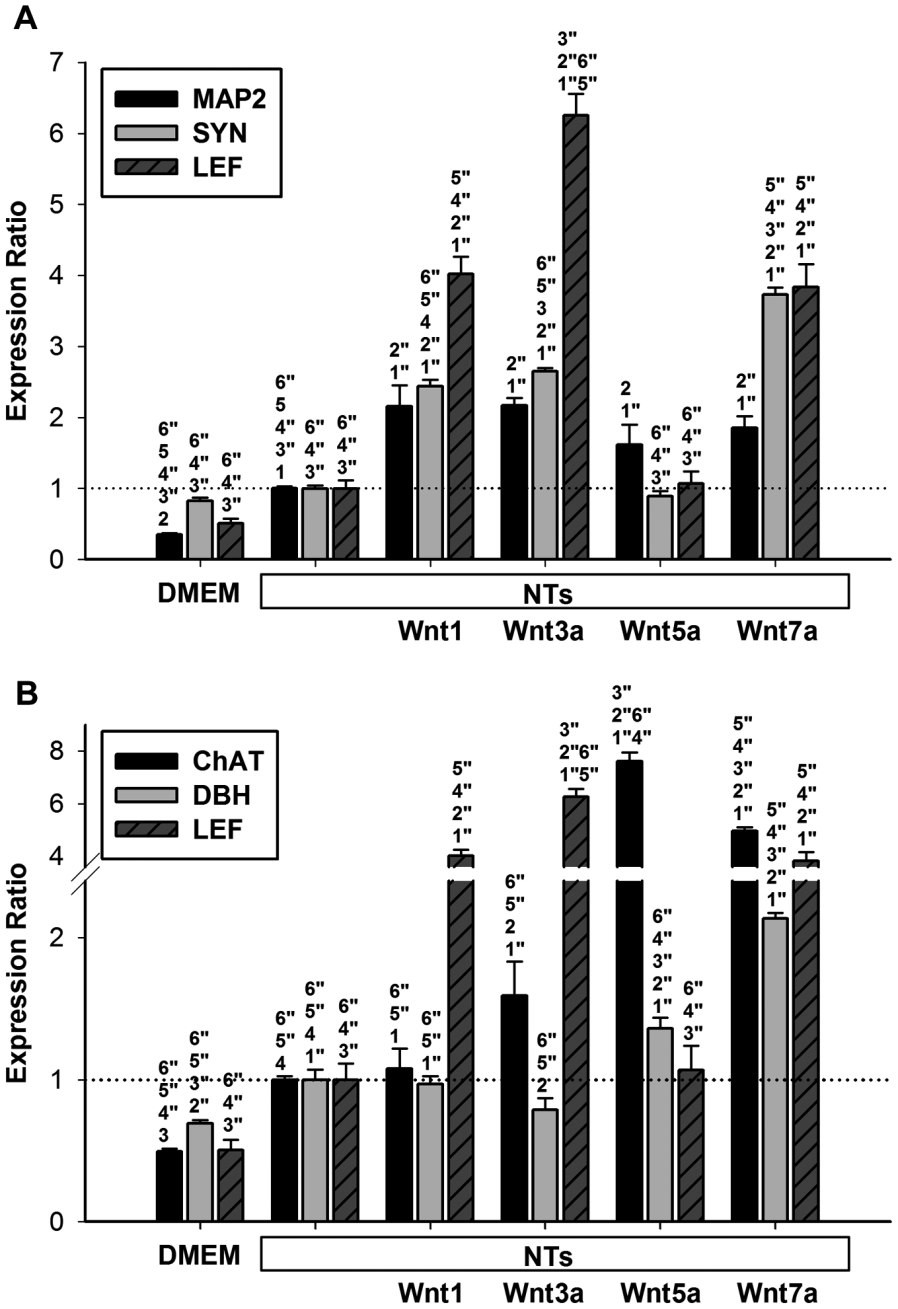
Neurogenic effects from different Wnts in NT-induced hMSCs. (A) mRNA levels of *MAP2*, *SYN1*, and *LEF1* were quantified after 48 h of different Wnt treatments (2 µg/ml) in hMSCs that had been treated with NTs for 1 week. All Wnts promoted *MAP2* expression, and Wnt7a induced the highest *SYN1* expression. Levels were normalized to those of NTs treatments (set to 1.0). Data are presented as the mean ± SD of one triplicate experiment that was representative of three independent experiments. ^1^ *p*<0.05, ^1″^ *p*<0.01 (DMEM vs. all groups); ^2^ *p*<0.05, ^2″^ *p*<0.01 (NTs vs. all groups); ^3^ *p*<0.05, ^3″^ *p*<0.01 (Wnt1 vs. all groups); ^4^ *p*<0.05, ^4″^ *p*<0.01 (Wnt3a vs. all groups); ^5^ *p*<0.05, ^5″^ *p*<0.01 (Wnt5a vs. all groups); ^6^ *p*<0.05, ^6″^ *p*<0.01 (Wnt7a vs. all groups). (B) mRNA levels of *ChAT*, *DBH*, and *LEF1* were quantified after 48 h of different Wnt treatments (2 µg/ml) in hMSCs that had been treated with NTs for 1 week. Wnt1 had no effects on *ChAT* or *DBH* expressions, but Wnt7a significantly induced both genes. Levels were normalized to those of NTs treatments (set to 1.0). Data are presented as the mean ± SD of one triplicate experiment that was representative of three independent experiments. ^1^ *p*<0.05, ^1″^ *p*<0.01 (DMEM vs. all groups); ^2^ *p*<0.05, ^2″^ *p*<0.01 (NTs vs. all groups); ^3^ *p*<0.05, ^3″^ *p*<0.01 (Wnt1 vs. all groups); ^4^ *p*<0.05, ^4″^ *p*<0.01 (Wnt3a vs. all groups); ^5^ *p*<0.05, ^5″^ *p*<0.01 (Wnt5a vs. all groups); ^6^ *p*<0.05, ^6″^ *p*<0.01 (Wnt7a vs. all groups).

Previous studies have reported that Wnts induce osteogenic differentiation [26], [27], [28]; therefore, to avoid osteogenic activation by Wnts, NTs were treated before Wnts were added to the culture medium. To determine which Wnt triggers the neurogenic differentiation of hMSCs, we added 2 µg/mL of hrWnt to hMSCs for 2 days after 1 week of treatment with NTs. For the general neuron marker, *MAP2*, all 4 Wnts induced approximately 2-fold increases compared with the NT group (Fig. 2A). No statistically significant changes in *MAP2* expression were observed among all 4 Wnt groups. To determine the function of synapses in neuronal differentiation, we examined the mRNA expression of *SYN1*, which was associated with the cytoplasmic surfaces of synaptic vesicles. qPCR data indicated that, when the cells were treated only with NTs, the expression of the synaptic marker *SYN1* did not change in cells (Fig. 2A). Wnt7a increased the expression of *SYN1* 3.7-fold and that of Wnt1 and Wnt3a 2.4- and 2.7-fold, respectively. However, Wnt5a did not significantly change *SYN1* expression compared with the NT only group (Fig. 2A).

To determine the differentiation of the hMSCs into specific neurons, we evaluated a marker of cholinergic neurons, *ChAT*, and a marker of dopaminergic neurons, *DBH*. Wnt3a, Wnt5a, and Wnt7a increased the mRNA levels of *ChAT* approximately 1.5-fold, 8-fold, and 5-fold, respectively, except Wnt1 exerted no effect on *ChAT* (Fig. 2B). Wnt5a and Wnt7a increased the mRNA levels of *DBH* approximately 1.2-fold and 2-fold, respectively; however, neither Wnt1 nor Wnt3a affected mRNA levels of *DBH* (Fig. 2B). These data indicated that Wnt7a can facilitate the results of neuronal differentiation in MSCs.

To determine the correlation of neuronal genes with Wnt signaling activity, we examined the expression of *LEF1*, a downstream target gene of canonical Wnt signaling [29] in the canonical Wnt/β-catenin pathway. Wnt1, Wnt3a, and Wnt7a markedly increased *LEF1* expression. As we hypothesized, a positive correlation of *SYN1* expression with the activation of the canonical Wnt/β-catenin pathway was observed (Fig. 2A). In contrast, *LEF1* exhibited no or minimal correlation with *ChAT* or *DBH*, which are markers of specific neuronal gene expression (Fig. 2B). These results suggested that more than one non-canonical signaling pathway controls specific neuronal gene expression.

Figures 1 and 2 show that NT-induced Wnt1 and Wnt7a can trigger *MAP2* expression in NT-treated hMSCs. Wnt1 exhibited no effects on specific neuronal differentiation, whereas Wnt7a enabled extensive triggering of neuronal differentiation in hMSCs. Therefore, we used Wnt7a in subsequent experiments.

### Dose- and time-dependent effects of Wnt7a treatment in neurotrophin-induced human mesenchymal stem cells

**Figure 3:**
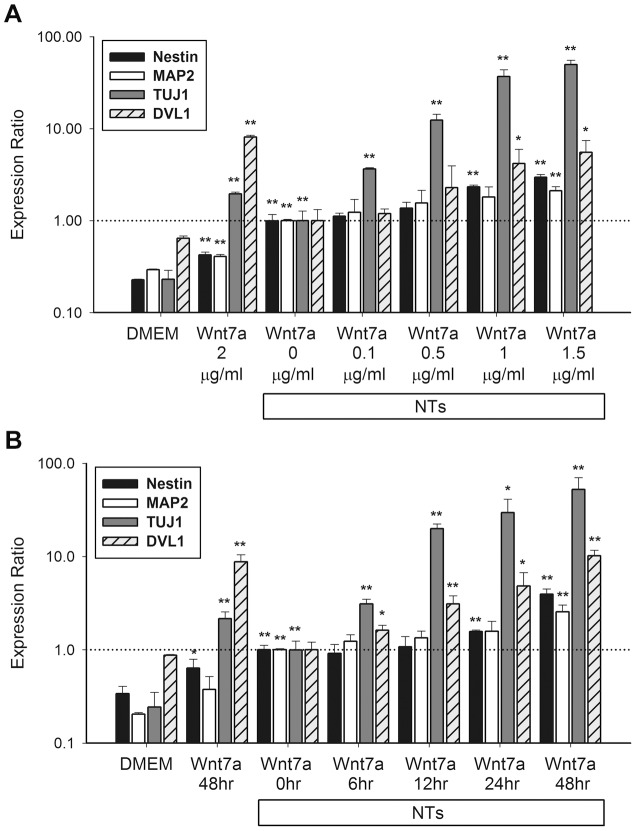
Dose- and time-dependent effects of Wnt7a in neurogenic hMSCs. mRNA levels of *nestin*, *MAP2*, *neurotubulin*, and *DVL1* were quantified after 48 h of Wnt7a treatment (0.1∼1.5 µg/ml) in a dose-dependent study (A) and at various times (i.e., 6∼48 h) after Wnt7a treatment (2 µg/ml) in a time-dependent study (B). NTs combined with Wnt7a promoted expression of neuronal genes related to the canonical Wnt pathway in time- and dose-dependent manners. Levels were normalized to those of NTs treatments (set to 1.0). Data are presented as the mean ± SD of one triplicate experiment that was representative of three independent experiments. * *p*<0.05, ** *p*<0.01 (NTs and Wnt7a vs. DMEM; NTs+Wnt7a vs. NTs).

To investigate the effects of Wnt7a on the neurogenesis of hMSCs, we added human recombinant Wnt7a to NT-induced hMSCs and subsequently analyzed the mRNA expression of neuronal markers. The expression of *nestin* (a neural progenitor marker), *neurotubulin* (a neuron-specific β3 tubulin), and *MAP2* (a general neuron marker) increased in a Wnt7a-dose- and time-dependent manner in the NT-induced cells (Fig. 3A, B). In addition, Wnt7a exhibited dose- and time-dependent positive effects on expression of the glial fibrillary acidic protein (an astrocyte marker) and myelin basic protein (an oligodendrocyte marker) in NT-treated hMSCs (Fig. S2).

DVL, a cytoplasmic phosphoprotein, was reported to act directly downstream of frizzled receptors. Expression of DVL1 exhibited a strong correlation with canonical Wnt signaling activation [30]. We examined whether Wnt7a-upregulated neurogenic differentiation involves canonical Wnt signaling. *DVL1* expression significantly increased in Wnt7a-induced hMSCs (Fig. 3A, B), indicating that Wnt7a enhances NT-induced neurogenesis in hMSCs through the canonical Wnt pathway.

### Expression of synapse markers is stimulated by Wnt7a in neurogenic human mesenchymal stem cells through the canonical Wnt pathway

**Figure 4:**
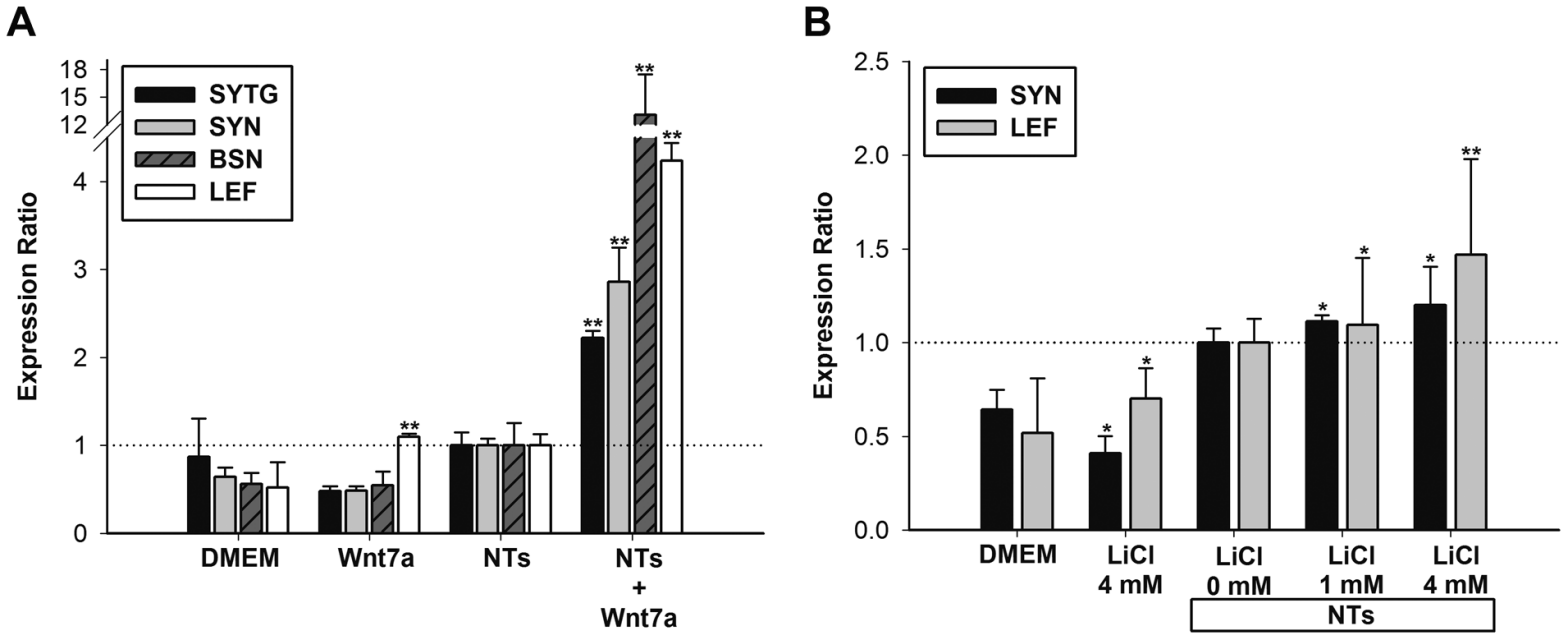
Induction of synaptic markers by Wnt7a and lithium in NT-stimulated hMSCs. (A) Wnt7a groups were treated with Wnt7a (2 µg/ml) for 2 days, and the NTs groups were treated with NTs for 9 days. After NTs treatment for 1 week, Wnt7a (2 µg/ml) was added to the NTs+Wnt7a groups for 2 days. mRNA levels of *LEF1*, *SYN1*, *BSN*, and *SYTG* were examined in hMSCs, and levels were normalized to those in the NTs control (set to 1.0). Wnt7a induced mRNA expressions of *SYN1*, *BSN*, and *SYTG* in NT-stimulated hMSCs, and this induction was related to upregulation of *LEF1*. Data are presented as the mean ± SD of one triplicate experiment that was representative of three independent experiments. * *p*<0.05, ** *p*<0.01 (NTs and Wnt7a vs. DMEM; NTs+Wnt7a vs. NTs). (B) LiCl groups were treated with LiCl (4 mM) for 2 days, and NTs groups were treated with NTs for 9 days. After NTs treatment for 1 week, LiCl (1 or 4 mM) was added to the NTs+LiCl groups for 2 days, mRNA levels of *SYN1* and *LEF1* were examined in hMSCs, and their levels were normalized to those in the DMEM control (set to 1.0). * *p*<0.05, ** *p*<0.01 (NTs and LiCl vs. DMEM; NTs+LiCl vs. NTs).

To determine whether Wnt7a triggers hMSCs to differentiate into neuron-like cells, we examined the expression of synaptic markers, namely *SYN1*, basson (*BSN*), and synaptotagmin (*SYTG*), in NT-treated hMSCs. Treatment with either NTs for 9 days or Wnt7a for 2 days produced no effects on *SYN1* expression in hMSCs (Fig. 4A). By contrast, following 7 days of pretreatment with NTs, hrWnt7a significantly increased *SYN1* mRNA 4-fold compared with the NT treatment alone (Fig. 4A). *LEF1* exhibited higher expression in the group treated with NTs and Wnt7a. In addition, NTs and Wnt7a triggered increases in *BSN* (approximately 12-fold) and *SYTG* (approximately 2-fold) in a similar manner.

**Figure 5:**
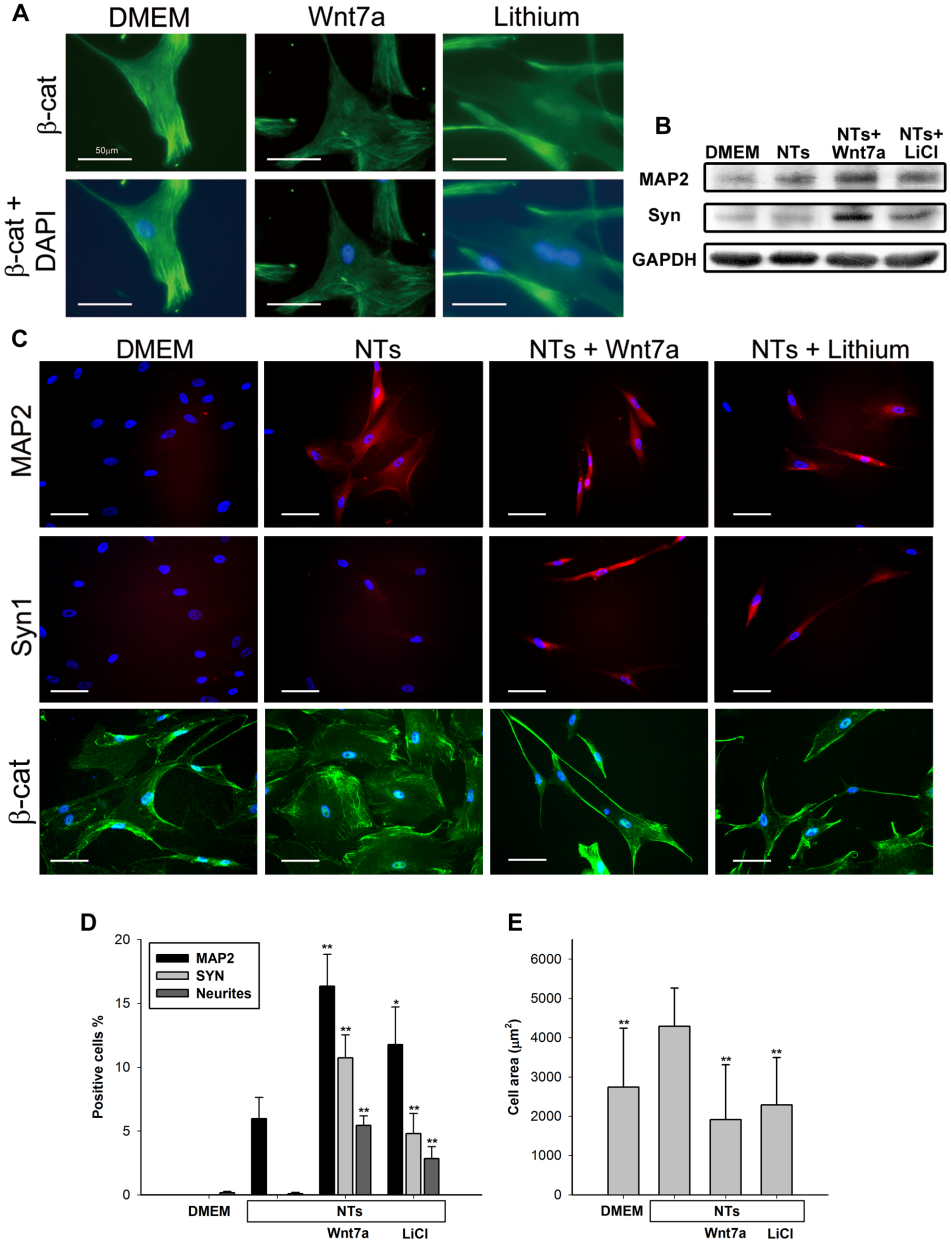
Immunostaining and immunoblotting of NT-stimulated hMSCs with Wnt7a or lithium. (A) p4 NT-treated hMSCs were stained with β-catenin (green). The NTs+Wnt7a groups were treated with NTs+Wnt7a for 24 h, and the NTs+lithium groups were treated with NTs+lithium for 24 h. 4,6-Diamidino-2-phenylindole (DAPI) (blue) was used as a counterstain. (B) p4 NT-treated hMSCs were immunoblotted with MAP2, SYN1, and GAPDH. The NT group was treated with NTs for 14 days. The NTs+Wnt7a and NTs+lithium groups were treated with NTs for 7 days first, and then with NTs+Wnt7a or lithium for 7 days. DMEM groups served as controls. (C) p4 NT-treated hMSCs were stained with MAP2 (red), SYN1 (red), and β-catenin (green). NTs groups were treated with NTs for 14 days. The NTs+Wnt7a and NTs+lithium groups were treated with NTs for 7 days first, and then NTs+Wnt7a or lithium for 7 days. DAPI (blue) was used as a counterstain. DMEM groups served as the control. The white bar represents 50 µm. (D) Percentages of MAP2-positive cells, SYN1-positive cells, and neurite-positive cells among all DAPI-positive cells. All data are presented as the mean ± SD. * *p*<0.05, ** *p*<0.01 (all vs. NTs). (E) Cell areas were calculated by β-catenin-positive cells from (B).

Immunocytochemistry and immunoblotting were conducted to confirm the results of qPCR analysis. We first observed β-catenin accumulation in nuclei after 24 h of treatment with Wnt7a or lithium. β-Catenin was colocalized with nuclear staining in Wnt7a- and lithium-treated hMSCs, whereas β-catenin accumulation was undetectable in the nuclei of the hMSC control group (Fig. 5A). This observation indicated that Wnt activation occurs via a canonical pathway. Immunocytochemistry and immunoblotting were conducted 7 days after the cells were treated with NTs for 7 days. Immunoblotting revealed that Wnt7a and lithium upregulated MAP2 and SYN1 expression; this result was consistent with that obtained using qPCR (Fig. 5B). Immunocytochemistry revealed that the NT group exhibited mild MAP2 expression (6%) but no SYN1 expression (Fig. 5C, D). By contrast, when Wnt7a or lithium was added to the culture medium with NTs for 7 days, hMSCs robustly expressed MAP2 (16.3% and 11.8%) and SYN1 (10.7% and 4.8%) (Fig. 5C, D). Morphological changes were evaluated using cytoskeletal β-catenin staining to quantitate the numbers of cell areas and neurite-positive cells. The immunocytochemical evaluation revealed that Wnt7a and lithium facilitated increases in neurite formation (5.4% and 2.8%) and decreases in cell bodies in NT-treated hMSCs after 14 days of treatment (Fig. 5D, E). The immunocytochemical evaluation revealed that Wnt7a triggered NT-treated hMSCs to differentiate into neuron-like cells through a canonical Wnt pathway.

### Induction of synapsin-1 expression in neurogenic human mesenchymal cells through the canonical Wnt pathway using a glycogen synthase kinase 3β inhibitor

To activate and mimic the canonical Wnt/β-catenin pathway, we used a GSK-3β inhibitor, lithium. The mRNA expression of *SYN1* induced by 1 and 4 mM LiCl and NTs was respectively 12.2% and 46.1% higher than those induced only by NTs (Fig. 4B). *LEF1* was activated in the lithium groups. This demonstrated that canonical Wnt signaling stimulated neuron-like cells generated by hMSCs.

### Inhibition of synapsin-1 expression in neurotrophin/Wnt7a-treated human mesenchymal stem cells by Wnt inhibitors and Wnt7a receptor antibodies

**Figure 6:**
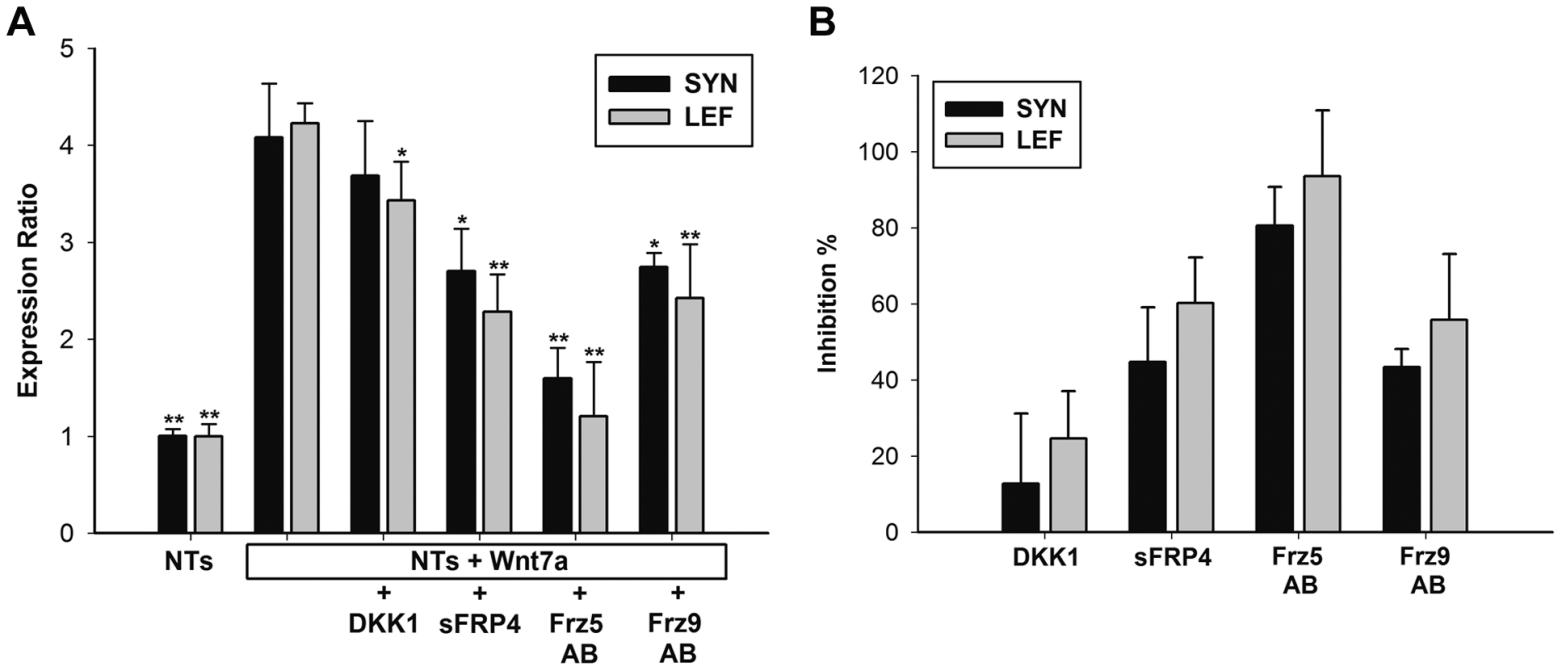
Inhibitory effects of Wnt inhibitors and blocking antibodies in Wnt7a-induced synapsin expression. (A) As described in "Materials and Methods", mRNA levels of *SYN1* and *LEF1* were examined by a qPCR. sFRP4 showed significant inhibition of *SYN1* expression, and Frz5 blocking antibodies greatly inhibited gene expressions. Levels were normalized to those in NTs groups (set to 1.0). * *p*<0.05, ** *p*<0.01 (NTs+Wnt7a vs. all groups). (B) Percentages of inhibition calculated from (A). Data are presented as the mean ± SD of one triplicate experiment that was representative of three independent experiments.

To determine whether *SYN1* induction is dependent on Wnt7a signaling in NT-treated hMSCs, we added Wnt7a inhibitors, the DKK1, sFRP4, Frz5, and Frz9 antibodies, to NT/Wnt7a-treated hMSCs. DKK1, a protein that interacts with LRP5/6, blocks the canonical Wnt pathway. Decreases in *SYN1* mRNA (12%) and *LEF1* levels (24%) occurred, as shown in Fig. 6A and 6B. sFRP, composed of a cysteine-rich Wnt-binding domain similar to the region in Frzs ligands, acts as a soluble antagonist for Wnt signals. A previous study demonstrated that sFRP4 bound to Wnt7a and inhibited canonical Wnt7a signaling in human endometrial cancer cells [31]. After the addition of sFRP4, the mRNA expression of *SYN1* (45%) and *LEF1* (60%) was inhibited in NT/Wnt7a-induced hMSCs (Fig. 6A, B). These results indicated that sFRP4 and DKK1 inhibited the activation of the canonical Wnt7a pathway and suppressed the differentiation of neuron-like cell generation by hMSCs. Recent studies have demonstrated that Frz5 and Frz9 act as Wnt7a receptors [31], [32]. Therefore, we used Frz5 and Frz9 antibodies as Wnt7a inhibitors. After incubating NT/Wnt7a-treated hMSCs with antibodies, *SYN1* expression decreased 81% and 43% in the Frz5 group and Frz9 group, respectively, compared with the control group (Fig. 6A, B). In addition, Frz5 (94%) and Frz9 (56%) antibodies suppressed *LEF1* expression, implying that Frz5 is a primary receptor that binds to Wnt7a, which activates the canonical Wnt pathway. These results indicated that Wnt7a interacts with Frz5 and Frz9 through the activation of the canonical Wnt pathway, controlling synapse formation by neurogenic hMSCs.

### Determination of specific neuron types by using Wnt7a in neurotrophin-induced human mesenchymal stem cells through the non-canonical/c-Jun N-terminal kinase pathway

**Figure 7:**
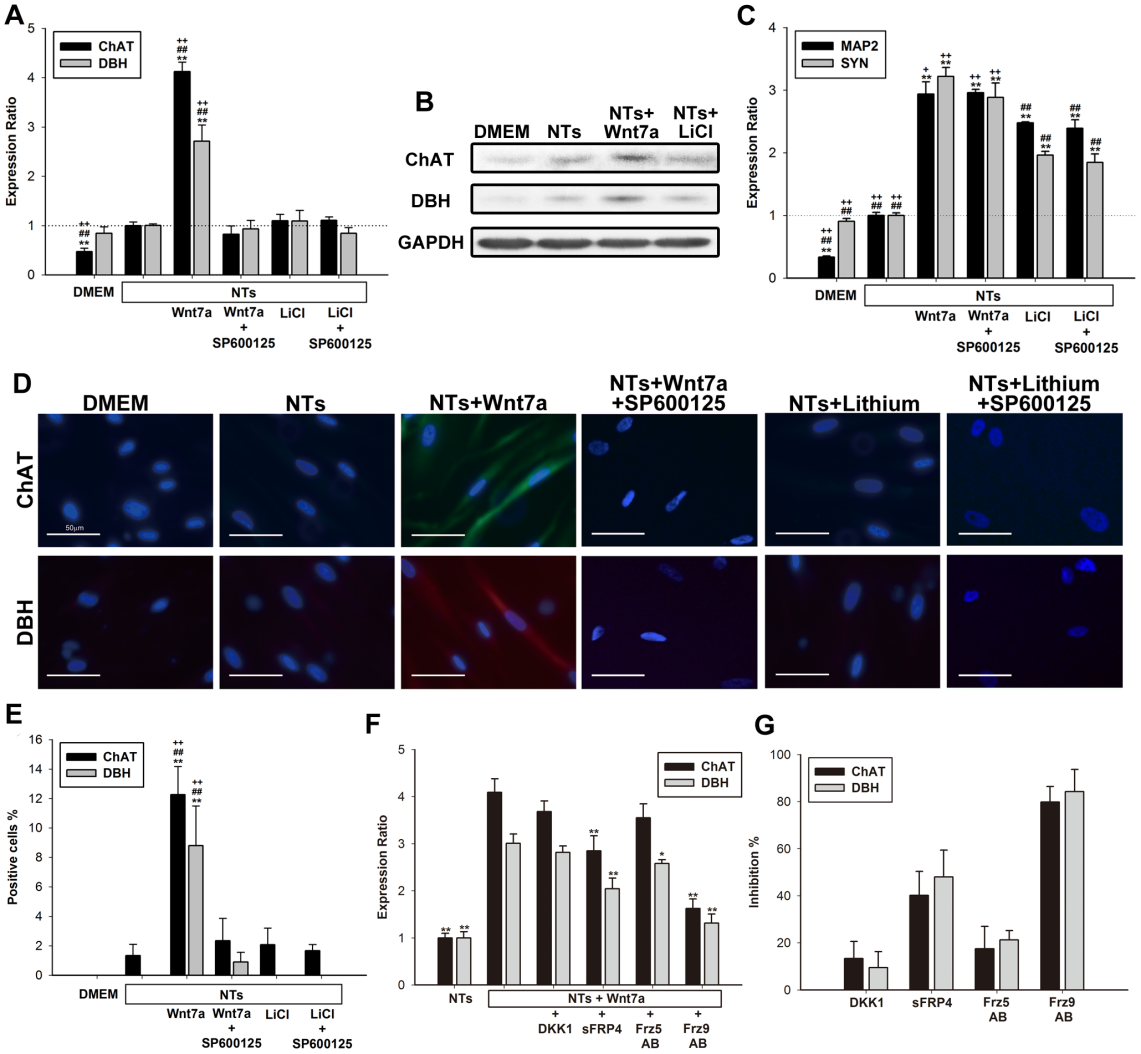
Neuronal specification by the non-canonical Wnt7a pathway in NT-induced hMSCs. (A) mRNA levels of *ChAT* and *DBH* were examined in NT-induced hMSCs, and SP600125 (15 µM) and Wnt7a (2 µg/ml) or LiCl (4 mM) were added to NT-induced hMSCs at the same time. Levels were normalized to those in the NTs control (set to 1.0). Wnt7a, but not lithium, stimulated mRNA levels in NT-induced hMSCs, and SP600125 totally inhibited Wnt7a-induced *ChAT* and *DBH* expressions. Data are presented as the mean ± SD of one triplicate experiment that was representative of the three independent experiments. * *p*<0.05, ** *p*<0.01 (all vs. NTs). ^#^ *p*<0.05, ^##^ *p*<0.01 (all vs. NTs+Wnt7a+SP600125). ^+^ *p*<0.05, ^++^ *p*<0.01 (all vs. NTs+LiCl+SP600125). (B) p4 NT-treated hMSCs were immunoblotted with ChAT, DBH, and GAPDH. The NT groups were treated with NTs for 14 days. The NTs+Wnt7a and NTs+lithium groups were treated with NTs for 7 days first, and then with NTs+Wnt7a or lithium for 7 days. DMEM groups served as controls. (C) Expression levels of *MAP2* and *SYN1* in NT-induced hMSCs with SP600125/Wnt7a or SP600125/LiCl are shown. SP600125 had no effect in *MAP2* or *SYN1* expression. Levels were normalized to those in NTs groups (set to 1.0). * *p*<0.05, ** *p*<0.01 (NTs vs. all groups). ^#^ *p*<0.05, ^##^ *p*<0.01 (all vs. NTs+Wnt7a+SP600125). ^+^ *p*<0.05, ^++^ *p*<0.01 (all vs. NTs+LiCl+SP600125). (D) p4 NT-treated hMSCs were stained with ChAT (green) and DBH (red). NTs groups were treated with NTs for 14 days. The NTs+Wnt7a and NTs+lithium groups were treated with NTs for the first 7 days and then with NTs+Wnt7a or lithium for the next 7 days. In inhibitory groups, SP600125 was added with Wnt7a or lithium in NT-induced hMSCs at the same time. DAPI (blue) was used as a counterstain. DMEM groups were used as controls. The white bar represents 50 µm. (E) Percentages of ChAT-positive cells and DBH-positive cells among all DAPI-positive cells calculated from (D). All data are presented as the mean ± SD. * *p*<0.05, ** *p*<0.01 (all vs. NTs). ^#^ *p*<0.05, ^##^ *p*<0.01 (all vs. NTs+Wnt7a+SP600125). ^+^ *p*<0.05, ^++^ *p*<0.01 (all vs. NTs+LiCl+SP600125). (F) As described in "Materials and Methods", mRNA levels of *ChAT* and *DBH* were examined by a qPCR. Levels were normalized to those in NTs groups (set to 1.0). * *p*<0.05, ** *p*<0.01 (NTs+Wnt7a vs. all groups). (G) Percentages of inhibition calculated from (E). Data are presented as the mean ± SD of one triplicate experiment that was representative of three independent experiments.

To examine whether Wnt7a signals trigger the differentiation of NT-treated hMSCs into specific neuron-like cells, we analyzed markers of cholinergic, dopaminergic, GABAergic, and serotonergic neurons in NT-treated hMSCs after the cells were treated with Wnt7a or lithium. Compared with the DMEM group, *DBH* mRNA was expressed at similar levels in NT-induced cells, whereas a 3-fold increase was observed after Wnt7a was added. Moreover, compared with the DMEM group, *ChAT*, glutamate decarboxylase-1 (*GAD*), and serotonin transporter (*SERT*) expression increased in the NT group, but treatment with Wnt7a and NTs induced an 8-fold increase in ChAT expression (Figs. 7A and S3). Treatment with lithium and NTs exerted no effect on the expression of *DBH*, *ChAT*, *GAD* and *SERT* compared with NT only group (Fig. 7A). Immunoblotting revealed features similar to those observed by conducting qPCR. Compared with the DMEM and NT groups, Wnt7a-induced increases in the protein levels of ChAT and DBH were greater than those induced by lithium (Fig. 7B). Previous studies have demonstrated that Wnt7a can trigger the canonical/β-catenin and JNK pathways [31], [33]. To determine whether Wnt7a triggers the non-canonical/JNK pathway to activate the differentiation of specific neurons, we added SP600125, a JNK inhibitor, and Wnt7a or lithium before conducting qPCR analysis. The results indicated that SP600125 completely inhibited Wnt7a-induced *ChAT* and *DBH* expression (Fig. 7A). However, SP600125 did not reduce Wnt7a-induced *MAP2* or *SYN1* expression (Fig. 7C). These findings indicate that Wnt7a induces the differentiation of specific neurons through the non-canonical/JNK pathway in NT-treated hMSCs.

Immunostaining revealed that NT/Wnt7a treatment induced 12.3% and 8.8% increases in ChAT and DBH expression in hMSCs; these results were consistent with those obtained in qPCR analysis. By contrast, decreases in ChAT (2.3%) and DBH (0.9%) were observed after SP600125 was added (Fig. 7D, E). In NT/lithium-treated hMSCs, no significant changes compared with the hMSCs treated only with NT were observed regardless of whether SP600125 was added (Fig. 7D, E). Overall, these results indicated that Wnt7a triggers immature neurons in hMSCs to differentiate into specific neuron-like cells, including cholinergic and dopaminergic neurons. In summary, our results indicated that the generation of neuron-like cells is triggered through both canonical and non-canonical Wnt pathways.

We then examined whether Wnt inhibitors and Frz blocking antibodies downregulate the non-canonical/JNK pathway. The results indicated that sFRP4 and Frz9 antibodies significantly lowered *ChAT* and *DBH* expression in Wnt7a-induced hMSCs (Fig. 7F). sFRP4 inhibited *ChAT* expression by 40% and *DBH* expression to 48%, and the Frz9 antibody significantly inhibited *ChAT* expression to 79% and *DBH* expression to 84% (Fig. 7G). DKK1 and the Frz5 antibody exerted mild inhibitory effects; therefore, they do not seem to play a major role in the non-canonical pathway (Fig. 7F, G). Collectively, these results indicated that Wnt7a activated the non-canonical/JNK pathway to induce the differentiation of specific neurons through the Frz9 receptor, but sFRP4 inhibited this induction.

## Discussion

Wnt signaling not only regulates embryonic development and adult homeostasis but also controls several processes in adult stem cells. Several previous studies have focused on regulatory mechanisms among Wnts and osteogenesis [28], chondrogenesis [34], adipogenesis [23], and myogenesis [35]. However, the relationship between Wnts and neurogenesis in hMSCs is unclear. Our results revealed that Wnt7a plays a crucial role in the specification and maturation of neurons from hMSCs. In addition, we proved that both the canonical and non-canonical Wnt signaling pathways facilitate neurogenesis triggering in hMSCs.

Takako et al. reported that hrWnt1 and Wnt3 (400 ng/mL) in a neural induction medium induced sensory neuron markers (*Ngn1*, *NeuroD*, *Brn3a*, and *P2X3*) and a glutamatergic neuron marker (*GluR 1-4*) in mouse MSCs via the canonical/β-catenin pathway [36]. NTs significantly stimulated hMSCs to express Wnt1 and Wnt7a. Both Wnts triggered more *MAP2* and *SYN1* expression than did NT treatment alone. However, Wnt1 exerted no effects on inducing the differentiation of cholinergic and dopaminergic neurons. Moreover, Wnt7a did not stimulate hMSCs to express the glutamatergic neuronal marker, glutamate dehydrogenase 1 (Fig. S3). Our results and those of Takako collectively indicate that Wnts play crucial roles in controlling MSC neurogenesis via canonical and non-canonical pathways.

Various methods trigger neurogenesis in hMSCs, including chemical induction [37], gene transfection [38], [39], and the use of conditioned media from rodent brains [39]. However, these methods are either limited in animal models or involve high risks. Cytokine induction of hMSCs appears to be safer than chemical induction or gene transfection in the human body. RA, BDNF, and NGF have been used as neurogenic stimulators of hMSCs [10]. We observed that NT/Wnt7a -treated hMSCs expressed axonal markers, such as SYN1. Our protocol for the neuronal differentiation of hMSCs is simple and feasible both *in vitro* and as an animal model for generating human neurons. Our protocol provides a new therapeutic opportunity for clinics.

Synaptic markers, such as SYN1, SYTG, BSN, synaptic vesicle 2 (SV2), and SYP, are markers of mature neurons. Cho et al. demonstrated that SV2 and SYP are present following treatment with RA and interleukin-1α for 10–12 days in hMSCs [40]. In addition, Trzaska et al. [41] demonstrated that treatment with Shh, FGF8, and bFGF for 12 days triggered hMSCs to become SV2-positive cells. Our data are consistent with those of 2 previous studies and revealed the quantitative effects of cytokines. The expression of *SYP* mRNA was observed in RT-PCR analysis on the fourteenth day after small-interference neuronal restrictive silencing factor (*NRSF*) RNA was used [42]. Gene transfection research has become more concerned with clinical application. Our protocol for neuronal induction is more efficient and feasible than other protocols. Our study clearly revealed that Wnt7a, after BDNF, NGF, and RA induction, functions as a synaptic enhancer.

Tuli et al. reported that human bone-marrow-derived MSCs incorporated with a chondrogenic factor, transforming growth factor (TGF)-β, express *Wnt7a* mRNA [34]. Zhou et al. demonstrated that TGF-β with lithium promoted chondrogenesis and inhibited adipogenesis [34], [43]. These results imply that Wnt7a is a chondrogenic factor in human bone-marrow-derived MSCs, although direct evidence is lacking. Our results indicated that Wnt7a facilitates neuronal differentiation in human bone-marrow-derived MSCs.

The Wnt family is divided into 2 groups based on the signaling pathways that they activate. Canonical Wnts, such as Wnt1 and Wnt3a, activate the canonical/β-catenin pathway, whereas non-canonical Wnts modulate β-catenin-independent signaling pathways, such as the Wnt/calcium and Wnt/JNK pathways [12]. In previous studies, synaptic formation and neurotransmitter releases in the brain have been controlled using Wnt7a through the Wnt canonical pathway [44], [45]. Wnt7b activates the canonical pathway to connect olfactory receptor neuron axons with the forebrain [46]. Furthermore, Wnt7b regulates dendritic development in hippocampal neurons through the Dvl, Rac, and JNK pathways [21]. In endometrial cancer cells, Wnt7a interacts with various receptors to stimulate the canonical Wnt pathway and Wnt/JNK pathway [31]. Our data indicated that both Wnt7a and lithium controlled SYN1 expression through the canonical Wnt pathway. However, Wnt7a, but not lithium, controlled neuronal determination in NT-induced hMSCs, and we used SP600125 to show that neuron-type differentiation is regulated by a Wnt-independent pathway. Our results revealed that Wnt7a triggered canonical Wnt signaling to differentiate general neurons and activated non-canonical Wnt signaling to transform hMSCs into cholinergic and dopaminergic neurons. Furthermore, we demonstrated that Wnt7a used different Frz receptors to determine activation of neuronal genes through a canonical or non-canonical pathway. Frz-determined activation is consistent with the findings of previous studies [31], [33].

Gene transfer is a method of converting hMSCs into mature neurons. In 2004, Dezawa et al. transfected hMSCs with the Notch intracellular domain and subsequently treated the hMSCs with bFGF, forskolin, and ciliary neurotrophic factors in media [38]. However, this protocol can generate only neural progenitor-like cells, and these cells require further glial-cell-line-derived neurotrophic factor treatment to mature. Recently, Yang et al. used siRNA to *NRSF*/repressor element-1 silencing transcription factors (*REST*) in hMSCs [42], and Park et al. used exogenous *Nurr1* gene delivery and electrical stimulation to induce the differentiation of hMSCs into nerve cells [47]. These methods induced rapid differentiation and caused hMSCs to develop a neuron-like morphology, but synapse formation and neuronal specification remained inadequately identified. From a clinical perspective, gene manipulations are complex and unpredictable regarding mature neuronal differentiation.

We noted Wnt7a was capable of enhancing neurogenic effect, which was based on the effects of NTs. Only Wnt7a had no neurogenic effects in hMSCs, although it activated signaling pathway. Using Wnt7a siRNA may provide the information whether NTs effects are originally from Wnt7a, and it does not completely answer our question. Our goal is to identify the effect of Wnt7a on NT-induced hMSCs, and is not to confirm neurogenic effects of NTs. In our results, NTs directs hMSCs toword the neuronal lineage, and NT-treated hMSCs can be further triggered by Wnt7a to a mature neuronal differentiation.

Transdifferentiation into different germ layers is controversial in developmental biology [48], [49]. In early 2010, Vierbuchen et al. demonstrated that functional neurons can be converted directly from mouse fibroblasts through ectopic expression of 3 transcription factors, *Ascl1*, *Brn2*, and *Myt1l* [50]. After mouse induced neurons (iNs) were generated, several research groups generated human iNs by using various sets of transcription factors in 2011 [51], [52], [53], [54], [55], [56]. This new direct reprograming method featuring defined factors indicates that transdifferentiation can occur across germ layers. Transdifferentiation can be controlled through epigenetic regulation and gene activation [57], [58]. In 2012, Ladewig et al. reported that inhibiting GSK-3β and SMAD signaling during reprogramming increased the efficiency of human iN generation as well as the purity of the resulting iNs [59]. Our results also supported the notion that the inhibition of GSK-3β plays a crucial role in neurogenic transdifferentiation. Although no molecular mechanisms of neurogenic transdifferentiation have been identified, our data clearly indicate that hMSCs are capable of neurogenic transdifferentiation.

Stem cell therapy is an alternative treatment for neural degeneration because the loss of dopaminergic neurons is a major cause of Parkinson's disease [60]. Trzaska et al. demonstrated that hMSCs differentiated into dopaminergic phenotypes following 12 days of incubation with Shh, FGF8, and bFGF [41]. In a subsequent study, they observed that this system facilitates the maturation of dopaminergic neurons after adding BDNF [61]. Our data revealed that Wnt7a can induce dopaminergic differentiation in hMSCs. In future research, we will evaluate the effects of combining Wnt7a with Shh and FGF to provide a possible translational application for Parkinson's disease.

Alzheimer's disease is characterized by damage to the cholinergic system of the basal forebrain [62], and the cholinergic differentiation of MSCs is poorly understood. Previous studies have reported that overexpression of the retinoblastoma family gene, RB, triggers cholinergic phenotypes in hMSCs [63]. In addition, one study reported that secreted amyloid precursor protein-α (sAPPα) promoted cholinergic differentiation in mouse MSCs [64]. Our study demonstrated that Wnt7a promotes cholinergic differentiation in hMSCs. Thus, combining Wnt7a with RB and sAPPα in hMSCs may enable cholinergic neurons to be generated more efficiently, providing a potential therapy for Alzheimer's disease.

**Figure 8:**
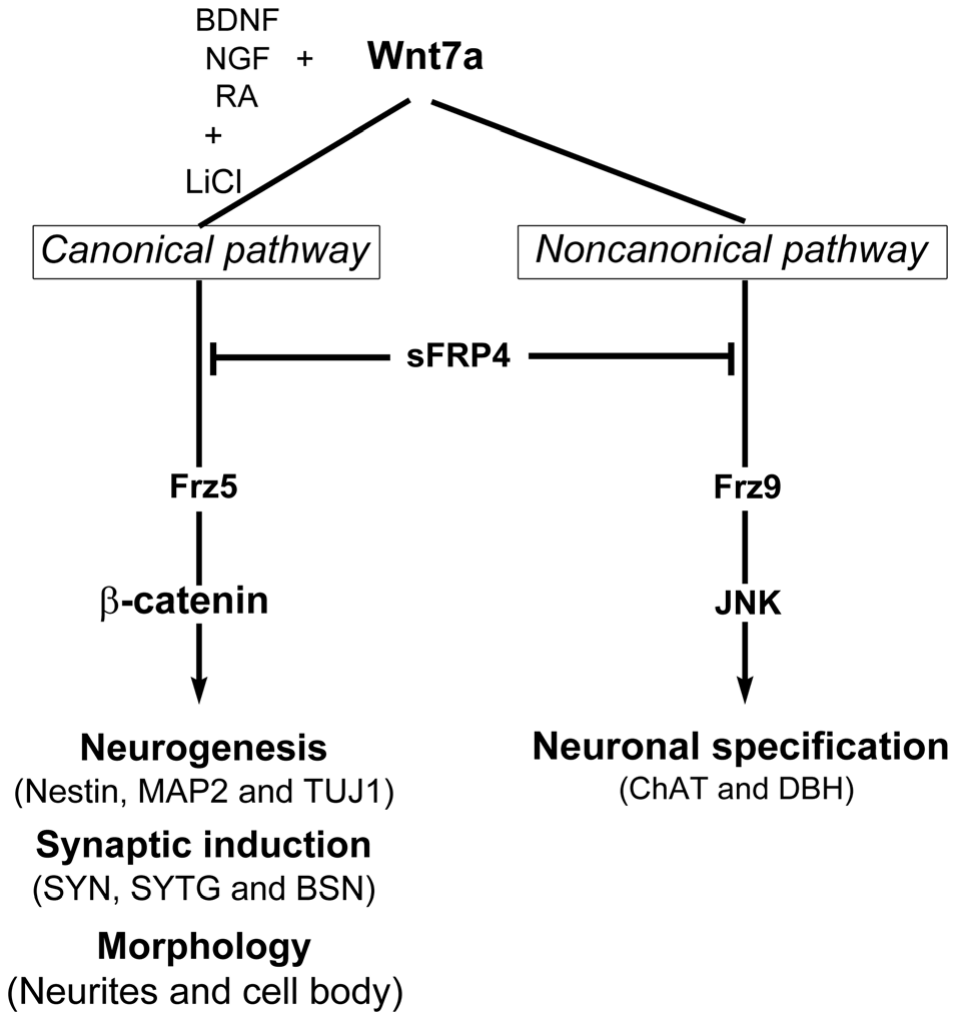
Summary of neuronal transdifferentiation regulation by Wnt7a in human bone marrow-derived MSCs. Accompanying NGF, BDNF, and RA, Wnt7a activated the canonical/β-catenin pathway via receptor Frz5 to promote neurogenesis and trigger synaptic marker (SYN1) expression. Furthermore, Wnt7a also triggered differentiations of cholinergic and dopaminergic neurons, but this effect was induced by another non-canonical/JNK pathway through Frz9 receptors. In this study, we showed that Wnt7a utilized two pathways to promote hMSC neurogenesis.

We demonstrated that Wnt7a promotes neuronal differentiation and induces synapse formation and neuronal phenotype determination through β-catenin-dependent or -independent pathways (Fig. 8). In conclusion, hrWnt7a enhances neuronal differentiation and neurite formation in hMSCs through various Frz receptors. These mechanisms may be employed widely in the transdifferentiation of other adult stem cells.

## Supporting Information

Figure S1 **Controls of immunocytochemistry.**
(TIF)

Figure S2 **Dose-dependent and time-dependent effects of GFAP and MBP expression in NT/Wnt7a-treated hMSCs.** Data are presented as the mean ± SD of one triplicate experiment that was representative of three independent experiments. * *p*<0.05, ** *p*<0.01 (NTs and Wnt7a vs. DMEM; NTs+Wnt7a vs. NTs).
(TIF)

Figure S3 **Expression of GAD, SERT and GLUD1 in NT/Wnt7a-treated hMSCs.** All data are presented as the mean ± SD. * *p*<0.05, ** *p*<0.01 (all vs. NTs).
(TIF)

## References

[1] Sanchez-Ramos JR (2002) Neural cells derived from adult bone marrow and umbilical cord blood. J Neurosci Res 69: 880–893. 12205681 10.1002/jnr.10337

[2] Wislet-Gendebien S, Hans G, Leprince P, Rigo JM, Moonen G, et al (2005) Plasticity of cultured mesenchymal stem cells: switch from nestin-positive to excitable neuron-like phenotype. Stem Cells 23: 392–402. 15749934 10.1634/stemcells.2004-0149

[3] Carvalho MM, Teixeira FG, Reis RL, Sousa N, Salgado AJ (2011) Mesenchymal stem cells in the umbilical cord: phenotypic characterization, secretome and applications in central nervous system regenerative medicine. Current stem cell research & therapy 6: 221–228. 21476975 10.2174/157488811796575332

[4] Ribeiro CA, Salgado AJ, Fraga JS, Silva NA, Reis RL, et al (2011) The secretome of bone marrow mesenchymal stem cells-conditioned media varies with time and drives a distinct effect on mature neurons and glial cells (primary cultures). Journal of tissue engineering and regenerative medicine 5: 668–672. 21774090 10.1002/term.365

[5] Munoz-Elias G, Marcus AJ, Coyne TM, Woodbury D, Black IB (2004) Adult bone marrow stromal cells in the embryonic brain: engraftment, migration, differentiation, and long-term survival. J Neurosci 24: 4585–4595. 15140930 10.1523/JNEUROSCI.5060-03.2004 PMC6729389

[6] Pittenger MF, Mackay AM, Beck SC, Jaiswal RK, Douglas R, et al (1999) Multilineage potential of adult human mesenchymal stem cells. Science 284: 143–147. 10102814 10.1126/science.284.5411.143

[7] Nemeth K, Leelahavanichkul A, Yuen PS, Mayer B, Parmelee A, et al (2009) Bone marrow stromal cells attenuate sepsis via prostaglandin E(2)-dependent reprogramming of host macrophages to increase their interleukin-10 production. Nature medicine 15: 42–49. 10.1038/nm.1905 PMC2706487 19098906

[8] Ren G, Zhang L, Zhao X, Xu G, Zhang Y, et al (2008) Mesenchymal stem cell-mediated immunosuppression occurs via concerted action of chemokines and nitric oxide. Cell Stem Cell 2: 141–150. 18371435 10.1016/j.stem.2007.11.014

[9] Rivera FJ, Couillard-Despres S, Pedre X, Ploetz S, Caioni M, et al (2006) Mesenchymal stem cells instruct oligodendrogenic fate decision on adult neural stem cells. Stem Cells 24: 2209–2219. 16763198 10.1634/stemcells.2005-0614

[10] Sanchez-Ramos J, Song S, Cardozo-Pelaez F, Hazzi C, Stedeford T, et al (2000) Adult bone marrow stromal cells differentiate into neural cells in vitro. Exp Neurol 164: 247–256. 10915564 10.1006/exnr.2000.7389

[11] Woodbury D, Schwarz EJ, Prockop DJ, Black IB (2000) Adult rat and human bone marrow stromal cells differentiate into neurons. J Neurosci Res 61: 364–370. 10931522 10.1002/1097-4547(20000815)61:4<364::AID-JNR2>3.0.CO;2-C

[12] Gordon MD, Nusse R (2006) Wnt signaling: multiple pathways, multiple receptors, and multiple transcription factors. J Biol Chem 281: 22429–22433. 16793760 10.1074/jbc.R600015200

[13] Shulman JM, Perrimon N, Axelrod JD (1998) Frizzled signaling and the developmental control of cell polarity. Trends in genetics: TIG 14: 452–458. 9825673 10.1016/s0168-9525(98)01584-4

[14] Yamanaka H, Moriguchi T, Masuyama N, Kusakabe M, Hanafusa H, et al (2002) JNK functions in the non-canonical Wnt pathway to regulate convergent extension movements in vertebrates. EMBO reports 3: 69–75. 11751577 10.1093/embo-reports/kvf008 PMC1083927

[15] Saneyoshi T, Kume S, Amasaki Y, Mikoshiba K (2002) The Wnt/calcium pathway activates NF-AT and promotes ventral cell fate in Xenopus embryos. Nature 417: 295–299. 12015605 10.1038/417295a

[16] Sheldahl LC, Slusarski DC, Pandur P, Miller JR, Kuhl M, et al (2003) Dishevelled activates Ca2+ flux, PKC, and CamKII in vertebrate embryos. The Journal of cell biology 161: 769–777. 12771126 10.1083/jcb.200211094 PMC2199364

[17] Castelo-Branco G, Rawal N, Arenas E (2004) GSK-3beta inhibition/beta-catenin stabilization in ventral midbrain precursors increases differentiation into dopamine neurons. J Cell Sci 117: 5731–5737. 15522889 10.1242/jcs.01505

[18] Hari L, Brault V, Kleber M, Lee HY, Ille F, et al (2002) Lineage-specific requirements of beta-catenin in neural crest development. J Cell Biol 159: 867–880. 12473692 10.1083/jcb.200209039 PMC2173383

[19] Krylova O, Herreros J, Cleverley KE, Ehler E, Henriquez JP, et al (2002) WNT-3, expressed by motoneurons, regulates terminal arborization of neurotrophin-3-responsive spinal sensory neurons. Neuron 35: 1043–1056. 12354395 10.1016/s0896-6273(02)00860-7

[20] Hirabayashi Y, Itoh Y, Tabata H, Nakajima K, Akiyama T, et al (2004) The Wnt/beta-catenin pathway directs neuronal differentiation of cortical neural precursor cells. Development 131: 2791–2801. 15142975 10.1242/dev.01165

[21] Ahmad-Annuar A, Ciani L, Simeonidis I, Herreros J, Fredj NB, et al (2006) Signaling across the synapse: a role for Wnt and Dishevelled in presynaptic assembly and neurotransmitter release. J Cell Biol 174: 127–139. 16818724 10.1083/jcb.200511054 PMC2064170

[22] Rosso SB, Sussman D, Wynshaw-Boris A, Salinas PC (2005) Wnt signaling through Dishevelled, Rac and JNK regulates dendritic development. Nat Neurosci 8: 34–42. 15608632 10.1038/nn1374

[23] Guo W, Flanagan J, Jasuja R, Kirkland J, Jiang L, et al (2008) The effects of myostatin on adipogenic differentiation of human bone marrow-derived mesenchymal stem cells are mediated through cross-communication between Smad3 and Wnt/beta-catenin signaling pathways. J Biol Chem 283: 9136–9145. 18203713 10.1074/jbc.M708968200 PMC2431017

[24] Clevers H (2006) Wnt/beta-catenin signaling in development and disease. Cell 127: 469–480. 17081971 10.1016/j.cell.2006.10.018

[25] Chamberlain G, Fox J, Ashton B, Middleton J (2007) Concise review: mesenchymal stem cells: their phenotype, differentiation capacity, immunological features, and potential for homing. Stem Cells 25: 2739–2749. 17656645 10.1634/stemcells.2007-0197

[26] Baksh D, Tuan RS (2007) Canonical and non-canonical Wnts differentially affect the development potential of primary isolate of human bone marrow mesenchymal stem cells. J Cell Physiol 212: 817–826. 17458904 10.1002/jcp.21080

[27] Gaur T, Lengner CJ, Hovhannisyan H, Bhat RA, Bodine PV, et al (2005) Canonical WNT signaling promotes osteogenesis by directly stimulating Runx2 gene expression. J Biol Chem 280: 33132–33140. 16043491 10.1074/jbc.M500608200

[28] Liu G, Vijayakumar S, Grumolato L, Arroyave R, Qiao H, et al (2009) Canonical Wnts function as potent regulators of osteogenesis by human mesenchymal stem cells. J Cell Biol 185: 67–75. 19349579 10.1083/jcb.200810137 PMC2700509

[29] Hovanes K, Li TW, Munguia JE, Truong T, Milovanovic T, et al (2001) Beta-catenin-sensitive isoforms of lymphoid enhancer factor-1 are selectively expressed in colon cancer. Nat Genet 28: 53–57. 11326276 10.1038/ng0501-53

[30] Nagahata T, Shimada T, Harada A, Nagai H, Onda M, et al (2003) Amplification, up-regulation and over-expression of DVL-1, the human counterpart of the Drosophila disheveled gene, in primary breast cancers. Cancer Sci 94: 515–518. 12824876 10.1111/j.1349-7006.2003.tb01475.x PMC11160156

[31] Carmon KS, Loose DS (2008) Secreted frizzled-related protein 4 regulates two Wnt7a signaling pathways and inhibits proliferation in endometrial cancer cells. Mol Cancer Res 6: 1017–1028. 18567805 10.1158/1541-7786.MCR-08-0039

[32] Winn RA, Van Scoyk M, Hammond M, Rodriguez K, Crossno JT Jr, et al (2006) Antitumorigenic effect of Wnt 7a and Fzd 9 in non-small cell lung cancer cells is mediated through ERK-5-dependent activation of peroxisome proliferator-activated receptor gamma. J Biol Chem 281: 26943–26950. 16835228 10.1074/jbc.M604145200

[33] Winn RA, Marek L, Han SY, Rodriguez K, Rodriguez N, et al (2005) Restoration of Wnt-7a expression reverses non-small cell lung cancer cellular transformation through frizzled-9-mediated growth inhibition and promotion of cell differentiation. The Journal of biological chemistry 280: 19625–19634. 15705594 10.1074/jbc.M409392200

[34] Tuli R, Tuli S, Nandi S, Huang X, Manner PA, et al (2003) Transforming growth factor-beta-mediated chondrogenesis of human mesenchymal progenitor cells involves N-cadherin and mitogen-activated protein kinase and Wnt signaling cross-talk. J Biol Chem 278: 41227–41236. 12893825 10.1074/jbc.M305312200

[35] Shang YC, Zhang C, Wang SH, Xiong F, Zhao CP, et al (2007) Activated beta-catenin induces myogenesis and inhibits adipogenesis in BM-derived mesenchymal stromal cells. Cytotherapy 9: 667–681. 17917885 10.1080/14653240701508437

[36] Kondo T, Matsuoka AJ, Shimomura A, Koehler KR, Chan RJ, et al (2011) Wnt signaling promotes neuronal differentiation from mesenchymal stem cells through activation of Tlx3. Stem cells 29: 836–846. 21374761 10.1002/stem.624 PMC3666870

[37] Barnabe GF, Schwindt TT, Calcagnotto ME, Motta FL, Martinez G Jr, et al (2009) Chemically-induced RAT mesenchymal stem cells adopt molecular properties of neuronal-like cells but do not have basic neuronal functional properties. PLoS One 4: e5222. 19370156 10.1371/journal.pone.0005222 PMC2667250

[38] Dezawa M, Kanno H, Hoshino M, Cho H, Matsumoto N, et al (2004) Specific induction of neuronal cells from bone marrow stromal cells and application for autologous transplantation. J Clin Invest 113: 1701–1710. 15199405 10.1172/JCI20935 PMC420509

[39] Yang CC, Shih YH, Ko MH, Hsu SY, Cheng H, et al (2008) Transplantation of human umbilical mesenchymal stem cells from Wharton's jelly after complete transection of the rat spinal cord. PLoS One 3: e3336. 18852872 10.1371/journal.pone.0003336 PMC2566594

[40] Cho KJ, Trzaska KA, Greco SJ, McArdle J, Wang FS, et al (2005) Neurons derived from human mesenchymal stem cells show synaptic transmission and can be induced to produce the neurotransmitter substance P by interleukin-1 alpha. Stem Cells 23: 383–391. 15749933 10.1634/stemcells.2004-0251

[41] Trzaska KA, Kuzhikandathil EV, Rameshwar P (2007) Specification of a dopaminergic phenotype from adult human mesenchymal stem cells. Stem Cells 25: 2797–2808. 17656644 10.1634/stemcells.2007-0212

[42] Yang Y, Li Y, Lv Y, Zhang S, Chen L, et al (2008) NRSF silencing induces neuronal differentiation of human mesenchymal stem cells. Exp Cell Res 314: 2257–2265. 18570921 10.1016/j.yexcr.2008.04.008

[43] Zhou S, Eid K, Glowacki J (2004) Cooperation between TGF-beta and Wnt pathways during chondrocyte and adipocyte differentiation of human marrow stromal cells. J Bone Miner Res 19: 463–470. 15040835 10.1359/JBMR.0301239

[44] Cerpa W, Godoy JA, Alfaro I, Farias GG, Metcalfe MJ, et al (2008) Wnt-7a modulates the synaptic vesicle cycle and synaptic transmission in hippocampal neurons. J Biol Chem 283: 5918–5927. 18096705 10.1074/jbc.M705943200

[45] Hall AC, Lucas FR, Salinas PC (2000) Axonal remodeling and synaptic differentiation in the cerebellum is regulated by WNT-7a signaling. Cell 100: 525–535. 10721990 10.1016/s0092-8674(00)80689-3

[46] Zaghetto AA, Paina S, Mantero S, Platonova N, Peretto P, et al (2007) Activation of the Wnt-beta catenin pathway in a cell population on the surface of the forebrain is essential for the establishment of olfactory axon connections. J Neurosci 27: 9757–9768. 17804636 10.1523/JNEUROSCI.0763-07.2007 PMC1986640

[47] Park JS, Yang HN, Woo DG, Jeon SY, Do HJ, et al (2012) Exogenous Nurr1 gene expression in electrically-stimulated human MSCs and the induction of neurogenesis. Biomaterials 33: 7300–7308. 22800541 10.1016/j.biomaterials.2012.06.069

[48] Chen Y, Teng FY, Tang BL (2006) Coaxing bone marrow stromal mesenchymal stem cells towards neuronal differentiation: progress and uncertainties. Cellular and molecular life sciences: CMLS 63: 1649–1657. 16786223 10.1007/s00018-006-6019-5 PMC11135999

[49] Croft AP, Przyborski SA (2006) Formation of neurons by non-neural adult stem cells: potential mechanism implicates an artifact of growth in culture. Stem cells 24: 1841–1851. 16868208 10.1634/stemcells.2005-0609

[50] Vierbuchen T, Ostermeier A, Pang ZP, Kokubu Y, Sudhof TC, et al (2010) Direct conversion of fibroblasts to functional neurons by defined factors. Nature 463: 1035–1041. 20107439 10.1038/nature08797 PMC2829121

[51] Pang ZP, Yang N, Vierbuchen T, Ostermeier A, Fuentes DR, et al (2011) Induction of human neuronal cells by defined transcription factors. Nature 476: 220–223. 21617644 10.1038/nature10202 PMC3159048

[52] Yoo AS, Sun AX, Li L, Shcheglovitov A, Portmann T, et al (2011) MicroRNA-mediated conversion of human fibroblasts to neurons. Nature 476: 228–231. 21753754 10.1038/nature10323 PMC3348862

[53] Ambasudhan R, Talantova M, Coleman R, Yuan X, Zhu S, et al (2011) Direct reprogramming of adult human fibroblasts to functional neurons under defined conditions. Cell stem cell 9: 113–118. 21802386 10.1016/j.stem.2011.07.002 PMC4567246

[54] Pfisterer U, Kirkeby A, Torper O, Wood J, Nelander J, et al (2011) Direct conversion of human fibroblasts to dopaminergic neurons. Proceedings of the National Academy of Sciences of the United States of America 108: 10343–10348. 21646515 10.1073/pnas.1105135108 PMC3121829

[55] Caiazzo M, Dell'Anno MT, Dvoretskova E, Lazarevic D, Taverna S, et al (2011) Direct generation of functional dopaminergic neurons from mouse and human fibroblasts. Nature 476: 224–227. 21725324 10.1038/nature10284

[56] Son EY, Ichida JK, Wainger BJ, Toma JS, Rafuse VF, et al (2011) Conversion of mouse and human fibroblasts into functional spinal motor neurons. Cell stem cell 9: 205–218. 21852222 10.1016/j.stem.2011.07.014 PMC3188987

[57] Yang N, Ng YH, Pang ZP, Sudhof TC, Wernig M (2011) Induced neuronal cells: how to make and define a neuron. Cell stem cell 9: 517–525. 22136927 10.1016/j.stem.2011.11.015 PMC4377331

[58] Vierbuchen T, Wernig M (2012) Molecular roadblocks for cellular reprogramming. Molecular cell 47: 827–838. 23020854 10.1016/j.molcel.2012.09.008 PMC3809030

[59] Ladewig J, Mertens J, Kesavan J, Doerr J, Poppe D, et al (2012) Small molecules enable highly efficient neuronal conversion of human fibroblasts. Nature methods 9: 575–578. 22484851 10.1038/nmeth.1972

[60] Schapira AH, Olanow CW (2004) Neuroprotection in Parkinson disease: mysteries, myths, and misconceptions. JAMA 291: 358–364. 14734599 10.1001/jama.291.3.358

[61] Trzaska KA, King CC, Li KY, Kuzhikandathil EV, Nowycky MC, et al (2009) Brain-derived neurotrophic factor facilitates maturation of mesenchymal stem cell-derived dopamine progenitors to functional neurons. J Neurochem 110: 1058–1069. 19493166 10.1111/j.1471-4159.2009.06201.x

[62] Whitehouse PJ, Struble RG, Hedreen JC, Clark AW, White CL, et al (1983) Neuroanatomical evidence for a cholinergic deficit in Alzheimer's disease. Psychopharmacol Bull 19: 437–440. 6635120

[63] Jori FP, Melone MA, Napolitano MA, Cipollaro M, Cascino A, et al (2005) RB and RB2/p130 genes demonstrate both specific and overlapping functions during the early steps of in vitro neural differentiation of marrow stromal stem cells. Cell Death Differ 12: 65–77. 15459751 10.1038/sj.cdd.4401499

[64] Chen CW, Boiteau RM, Lai WF, Barger SW, Cataldo AM (2006) sAPPalpha enhances the transdifferentiation of adult bone marrow progenitor cells to neuronal phenotypes. Curr Alzheimer Res 3: 63–70. 16472205 10.2174/156720506775697205

