# Supplementary figures and images for: Wnts Enhance Neurotrophin-Induced Neuronal Differentiation in Adult Bone-Marrow-Derived Mesenchymal Stem Cells via Canonical and Noncanonical Signaling Pathways
Source: PLoS One. 2014 Aug 29;9(8):e104937. doi: 10.1371/journal.pone.0104937 (PMC4149376; doi:10.1371/journal.pone.0104937)

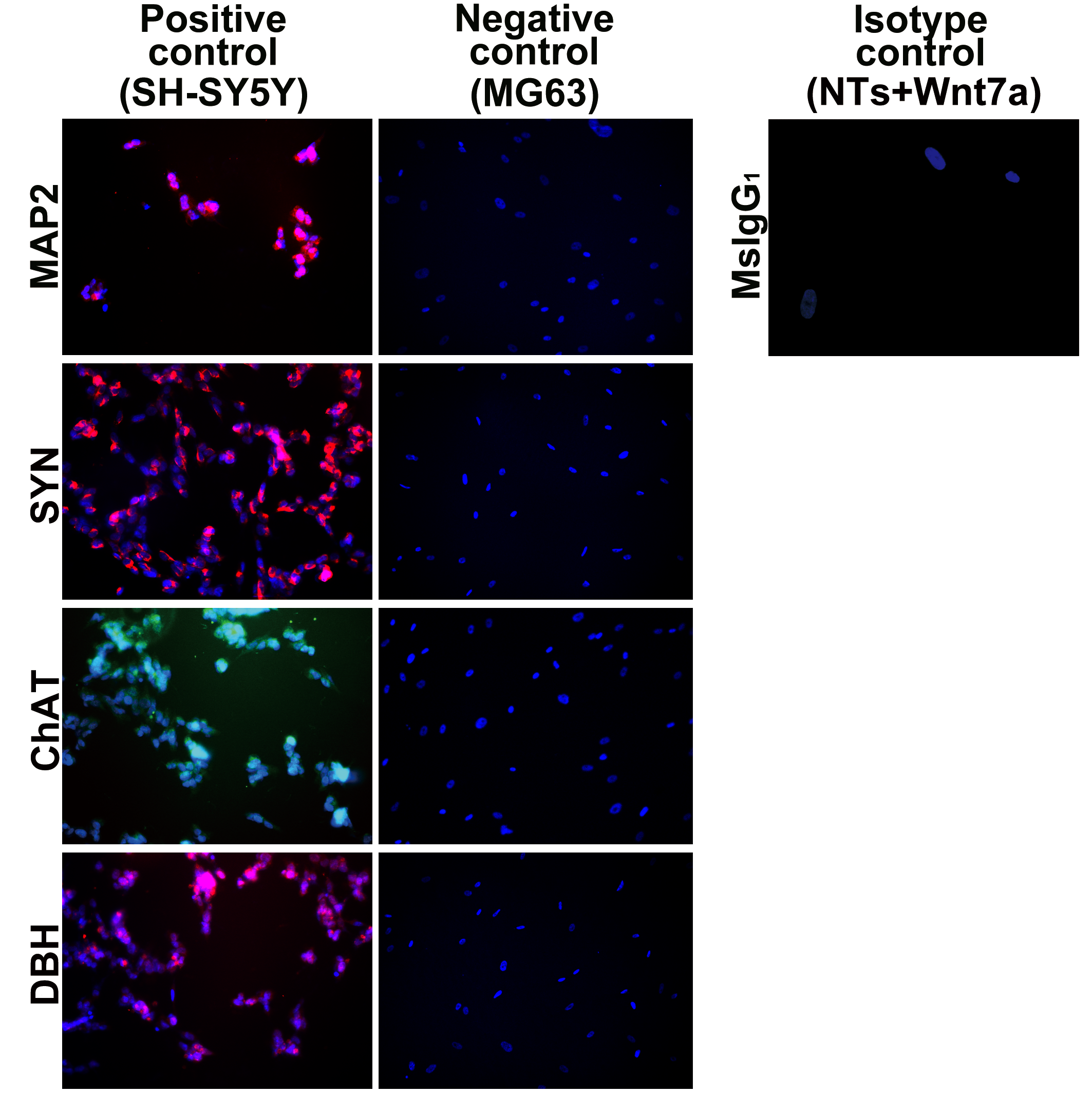

Supplement: Figure S1 — Controls of immunocytochemistry. (TIF) [file pone.0104937.s001.tif]

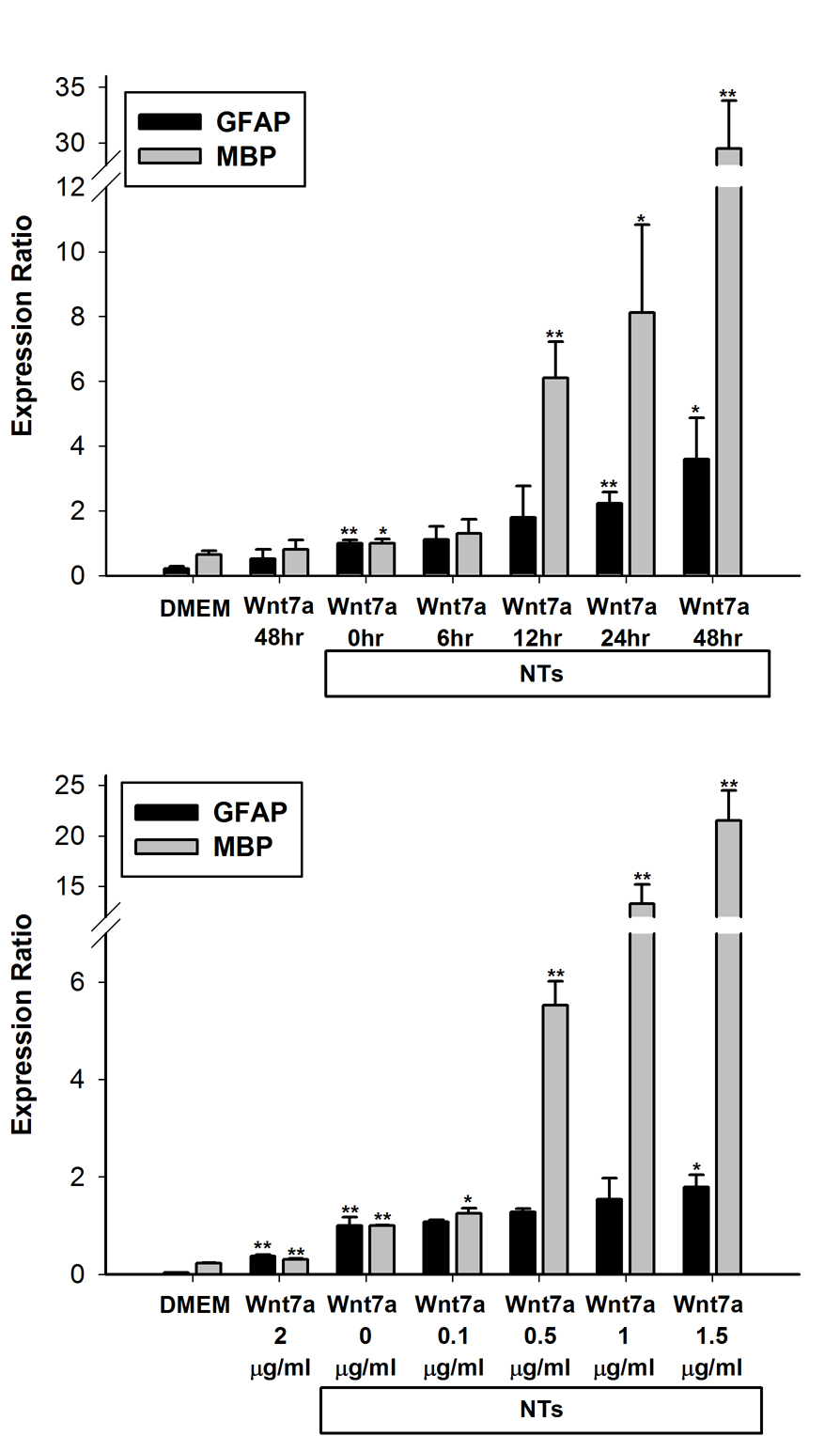

Supplement: Figure S2 — Dose-dependent and time-dependent effects of GFAP and MBP expression in NT/Wnt7a-treated hMSCs. Data are presented as the mean ± SD of one triplicate experiment that was representative of three independent experiments. * p<0.05, ** p<0.01 (NTs and Wnt7a vs. DMEM; NTs+Wnt7a vs. NTs). (TIF) [file pone.0104937.s002.tif]

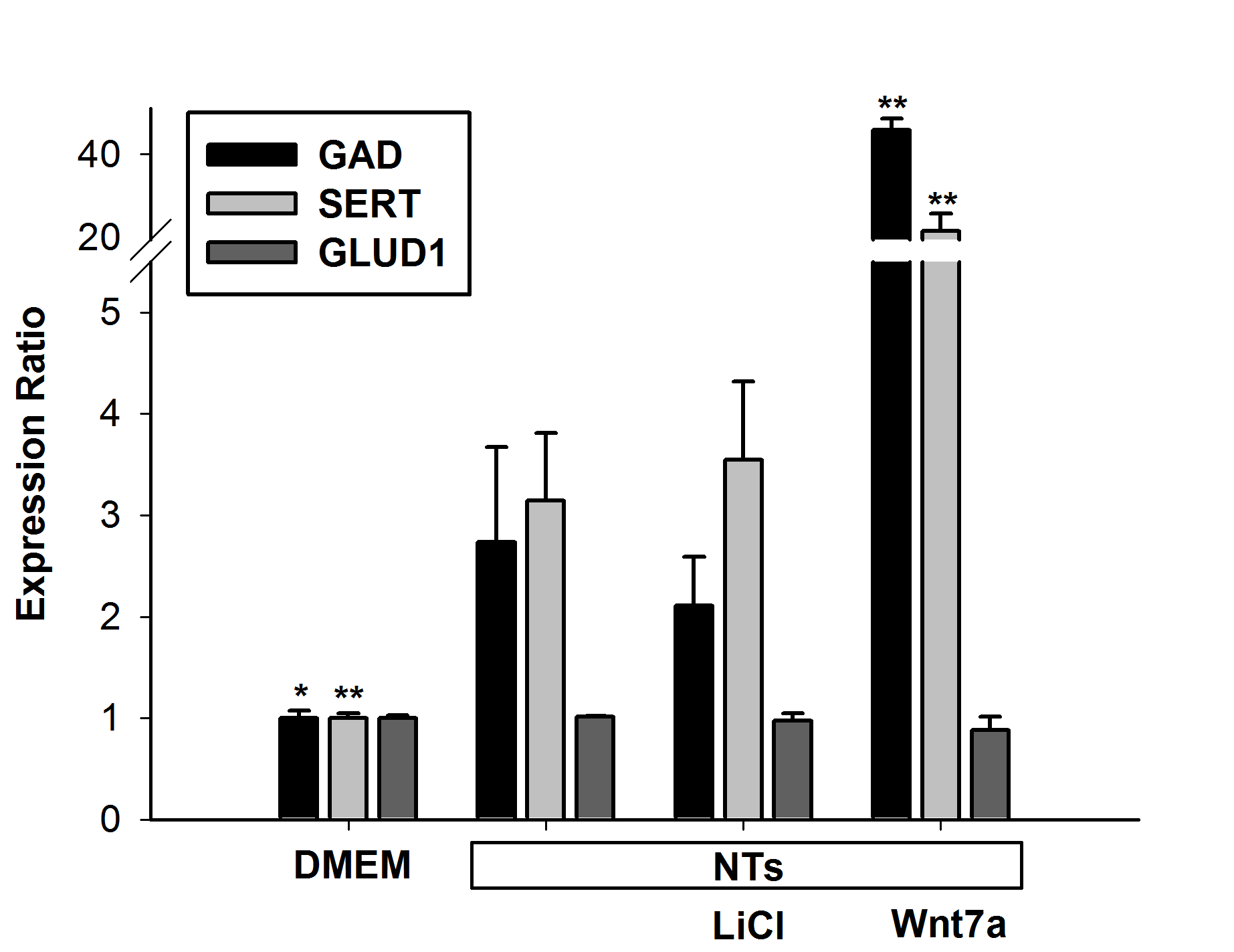

Supplement: Figure S3 — Expression of GAD, SERT and GLUD1 in NT/Wnt7a-treated hMSCs. All data are presented as the mean ± SD. * p<0.05, ** p<0.01 (all vs. NTs). (TIF) [file pone.0104937.s003.tif]
